# Latent mechanisms of language disorganization relate to specific dimensions of psychopathology

**DOI:** 10.1038/s44220-024-00351-w

**Published:** 2024-11-25

**Authors:** Isaac Fradkin, Rick A. Adams, Noam Siegelman, Rani Moran, Raymond J. Dolan

**Affiliations:** 1 Max Planck University College London Centre for Computational Psychiatry and Ageing Research, London, UK; 2 Department of Psychology, Hebrew University of Jerusalem, Jerusalem, Israel; 3 Centre for Medical Image Computing and AI, University College London, London, UK; 4 Department of Cognitive and Brain Sciences, Hebrew University of Jerusalem, Jerusalem, Israel; 5 School of Biological and Behavioural Sciences, Queen Mary University of London, London, UK; 6 Wellcome Trust Centre for Human Neuroimaging, University College London, London, UK

**Keywords:** Psychiatric disorders, Psychology, Language, Psychosis, Computational neuroscience

## Abstract

Comprehensible communication is critical for social functioning and well-being. In psychopathology, incoherent discourse is assumed to reflect disorganized thinking, which is classically linked to psychotic disorders. However, people do not express everything that comes to mind, rendering inferences from discourse to the underlying structure of thought challenging. Indeed, a range of psychopathologies are linked to self-reported disorganized thinking in the absence of language output incoherence. Here we combine natural language processing and computational modeling of free association to detail the relationship between disorganized thinking and language (in)coherence in a large sample of participants varying across different dimensions of psychopathology. Our approach allowed us to differentiate between disorganized thinking, disinhibited thought expression and deliberate creativity. We find evidence for both under-regulated and over-regulated disorganized thinking, which relate to two specific dimensions of psychopathology: self-reported eccentricity and suspiciousness. Broadly, these results underscore the theoretical progress afforded by analyzing latent dimensions underlying behavior and psychopathology.

## Abstract (web summary)

Using natural language processing and computational modeling, this study finds distinctions among disorganized thinking, disinhibition through expression and deliberate creativity, which are differentially associated with eccentricity and suspiciousness dimensions of psychopathology.

## Main

Communication relies on shared meaning. For example, when talking about a daily routine, we tend to think about things such as morning preparations, work and family time. An overlap in these associations fosters a sense of understanding and connection among interlocutors^1,2^. Occasionally, a topic can evoke atypical or random associations, which others find hard to understand. In such instances, effective communication depends on an ability to regulate expression of these associations.

In psychopathology, incoherent communication and the articulation of unusual associations is central to a diagnostic ascription of formal thought disorder (FTD; Box 1), a common phenotype in psychosis^3,4^. In line with the idea that coherent communication relies on an interaction between shared internal representations and the monitoring and regulation of speech, theories of FTD implicate impairments in both semantic memory and executive function^5–10^. Until now, these domains have been measured separately, leaving unresolved the question of how atypical associative thinking and executive dysregulation interact.

The importance of understanding disorganized thought dynamics separately from their linguistic expression is highlighted by evidence that these are not necessarily synonymous^11,12^. Indeed, people experiencing disorganized thinking can, in principle, mitigate negative social responses by regulating speech output^13^. Furthermore, an ability to control and regulate the outputs of underconstrained associative thinking may mark a line between incoherent communication and adaptive creative communication^14^. Finally, introspective experiences of disorganized thinking occur in a range of non-psychotic disorders (for example, obsessive–compulsive disorder and attention deficit hyperactivity disorder (ADHD))^15–18^. However, these conditions rarely show speech incoherence, leaving unresolved the question of whether these self-reported experiences reflect an actual disorganization of thinking. These considerations underline a need for an approach capable of capturing subtle, and even concealed (that is, filtered), thought disorganization.

In our study, we aimed to map alterations in thought and discourse across dimensions of psychopathology and detail their underlying mechanisms. Whereas recent evidence stresses the challenge of distinguishing different diagnostic categories based on linguistic features^19^, a dimensional approach to psychopathology, combined with computational modeling, is optimal for uncovering shared and unique mechanisms underlying partially overlapping phenotypes^20–22^.

Dimensions of psychopathology were derived based on participants’ self-reported symptoms and communication difficulties. Discourse incoherence was measured by analyzing participant-generated, speech-like free narratives, using established natural language processing (NLP) methods^23–27^. Analysis of free narratives can reveal even subtle alterations in language in psychopathology^28,29^ but cannot explain why they occur, nor provide evidence for concealed thought disorganization. For the latter, we used a separate free association task, which lends itself to computational modeling that can capture interactions between associative organization and executive regulation^30^.

**Fig. 1:**
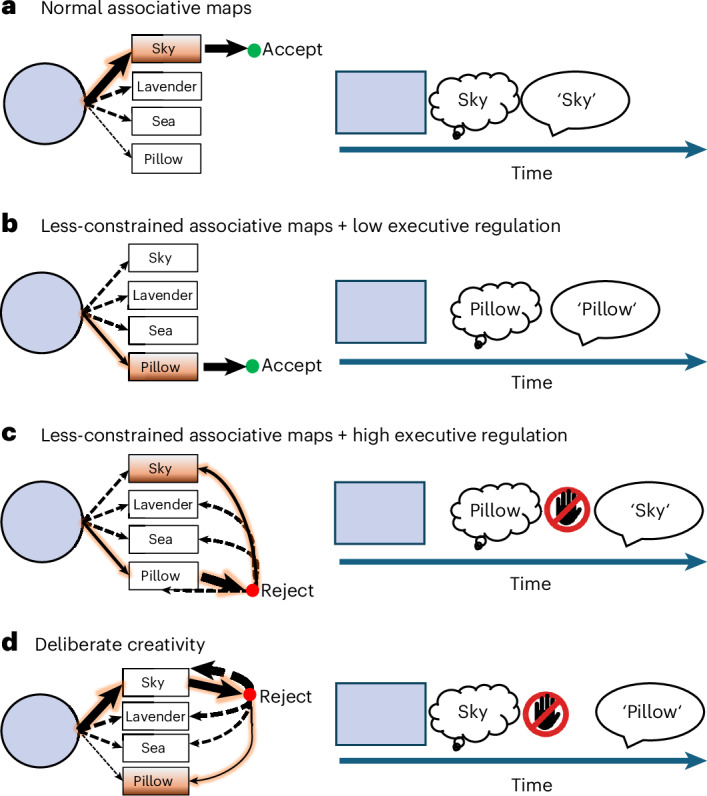
Proposed candidate mechanisms for loosening of associations using a color association task. Here, participants are required to report an association to a color (pale blue in this example). **a**–**c**, Disorganized associative thinking is formalized as less-constrained associative maps, increasing the probability of thinking an atypical association such as ‘pillow’ (**b** and **c**) rather than a more typical association to the depicted color, such as ‘sky’ (**a**). A secondary executive regulation mechanism can be used to inhibit the expression of such atypical associations (whether they are more likely to come to mind or not). Reduced regulation makes atypical associations more likely to be reported (**b**), whereas intact (or increased) regulation may result in covert thought disorder (**c**). **d**, Finally, increased reporting of atypical associations could also be explained by an increased motivation to express such ‘original’ associations. The dashed arrows represent possible associations, whereas solid arrows (with an orange frame) represent the actual enacted policy. Arrow width represents the probability of thinking a given association. These illustrative enacted policies are also depicted on a timeline, which shows how both executive regulation (illustrated by a crossed-out hand symbol) and weaker associations (for example, ‘pillow’) increase RTs.

We formalize associative disorganization as greater variability in the generation process, effectively increasing the probability that a weak association will come to mind. Executive regulation of thought expression is formalized as a tendency to avoid reporting certain (for example, atypical) associations. The semi-Markov process (SMP) model used here enables formalizing the interplay between these processes and inferring them based on the interaction between the types of association participants report and how quickly they report them (Fig. 1). For instance, concealed disorganized thinking will lead to slower, but typical, associations. The model also enables distinguishing atypical associations that reflect disorganized thinking from similar associations reflecting a deliberate attempt to report ‘creative’ and unconventional associations^31^.

Together, this combination of methodologies will enable characterizing the mechanisms underlying difficulties in communication in psychopathology, while determining whether these mechanisms are indeed transdiagnostic or confined to specific psychiatric dimensions. Given the ubiquity of transdiagnostic mechanisms in psychopathology^20,32^, finding diagnostic specificity despite casting a wide net encompassing subtle and even covert language alterations would be particularly revealing and informative.

Box 1 Incoherent language and communication in FTD FTD consists of different linguistic markers that together render speech incoherent, idiosyncratic and difficult to understand. These markers variously include a pattern of speech in which ideas only obliquely relate to one another, the inclusion of irrelevant details and a drift from addressing the original question or message. In some cases, the latter characteristics lead to completely incoherent speech (for example, a patient with schizophrenia was asked about his name said in response: “Well, let’s say you might have thought you had something from before, but you haven’t got it anymore”^70^), whereas in others they have a subtler impact. The latter is exemplified by the following response from a participant in this study when asked to describe the daily routine of an average person:

“That entirely depends on each individual. The routine of an army major will differ greatly from that of a whining student and both from a lady of 93. For the lady of ninety-three, the biggest surprise of the day is waking up at all. For everybody else the day starts with waking up. Then we get up, except usually the student who is too lazy and expects life to come to him and the elderly and infirm who have less choice in the matter anyway. Some people dress, some take a shower, some take a bath and some have overslept. This introduces a high degree of variety into the routine which also introduces the concepts of free will, individual responsibility, and opportunity, except for the student who rejects the existence of these concepts on principle. […]”

## Results

### Self-reported communication difficulties in psychopathology

**Fig. 2:**
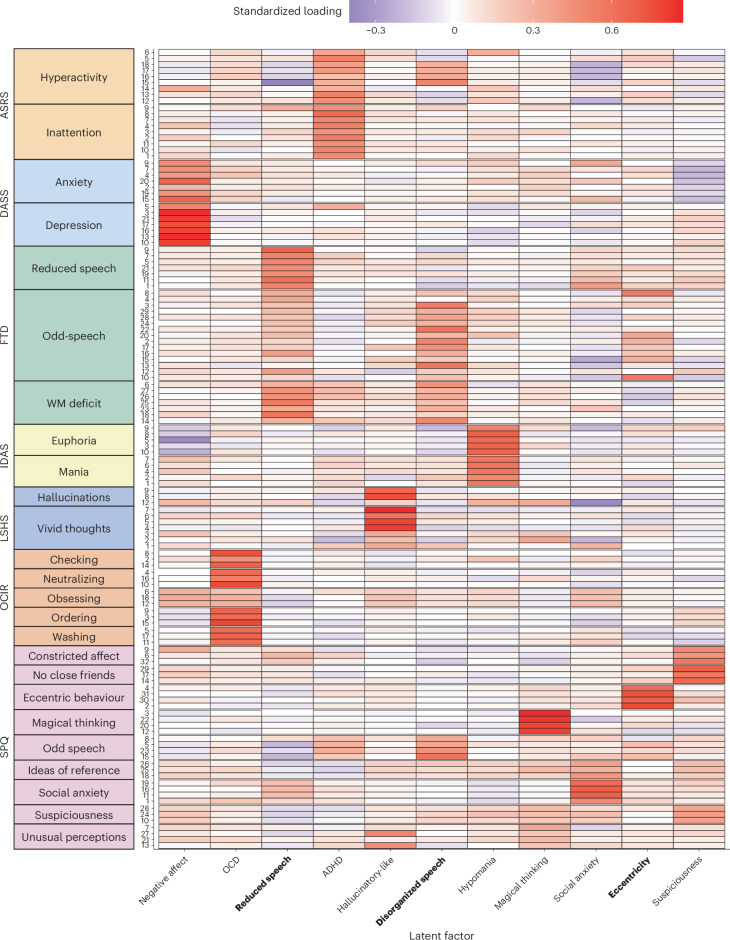
Dimensions of self-reported psychopathology. Standardized factor loadings for items from subject psychiatric questionnaires (*y* axis, organized by questionnaires, their original subscales and original item numbering) on 11 latent factors (*x* axis), 3 of which assess some form of self-reported abnormality in speech (highlighted in bold font). See Methods for the versions and references of the scales and questionnaires. ASRS, Adult ADHD Self-Report Scale; DASS, Depression and Anxiety Scale; IDAS, Inventory of Depression and Anxiety Symptoms; LSHS, Launay–Slade Hallucination Scale; OCD, obsessive–compulsive disorder; OCIR, Obsessive–Compulsive Inventory; SPQ, schizotypy questionnaire; WM, working memory.

**Extended Data Fig. 1:**
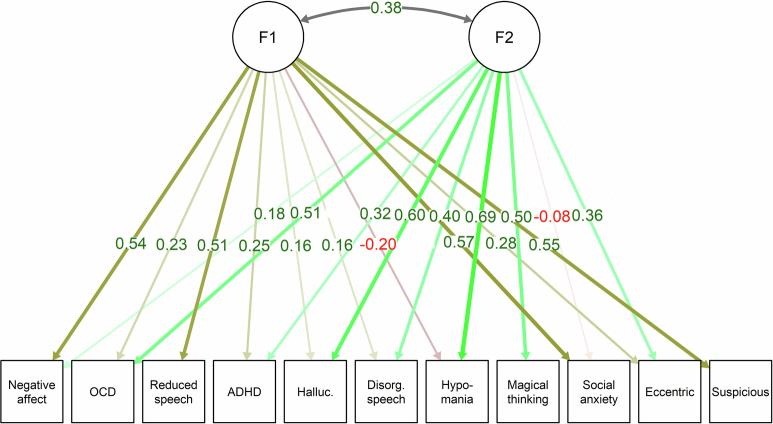
Higher-order factor structure. Higher-order factor analysis was conducted by first submitting the factor scores (depicted in Fig. 2) to an additional parallel analysis (using principal component analysis), which indicated that the first-order factors are best explained by two second-order factors. Then, maximum likelihood EFA, with Oblimin rotation was conducted to derive the higher-order factor structure and factor scores.

Participants (*N* = 1,000), recruited online, performed 2 tasks: 1 task measuring their use of language and 1 measuring their associative thinking. They also completed questionnaires measuring symptoms of different psychopathologies and self-reported communication difficulties. Exploratory factor analysis suggested the correlations between the 128 questions included in these questionnaires were best accounted for by 11 factors (Fig. 2), 3 of which included items explicitly measuring communication difficulties (FTD on the *y* axis of Fig. 2). First, a factor we refer to as ‘reduced speech’ included items pertaining to difficulty initializing or maintaining a conversational output (“My speech gets suddenly blocked”). Second, a factor we refer to as ‘disorganized speech’ included items measuring long-winded or poorly organized speech (“I go on beating about the bush instead of getting to the point of the conversation”). Finally, a factor we refer to as ‘eccentricity’ included items pertaining to perceived atypicality (by both self and others) in speech (“I use long and unusual words to say simple things”) and behavior. Notably, except for the disorganized speech dimension, which included some items from the ADHD questionnaire, self-reported communication difficulties mainly covaried with items measuring schizotypy. Whereas some of the resulting dimensions are relatively specific (compare refs. ^20,32^), we also extracted their higher-level structure to probe broader, transdiagnostic mechanisms. We found two higher-level dimensions pertaining to ‘internalizing symptoms’ (for example, negative affect and reduced speech) and ‘positive symptoms’ (for example, hypomania and disorganized speech; Extended Data Fig. 1).

### Atypicality and incoherence in language in psychopathology

**Fig. 3:**
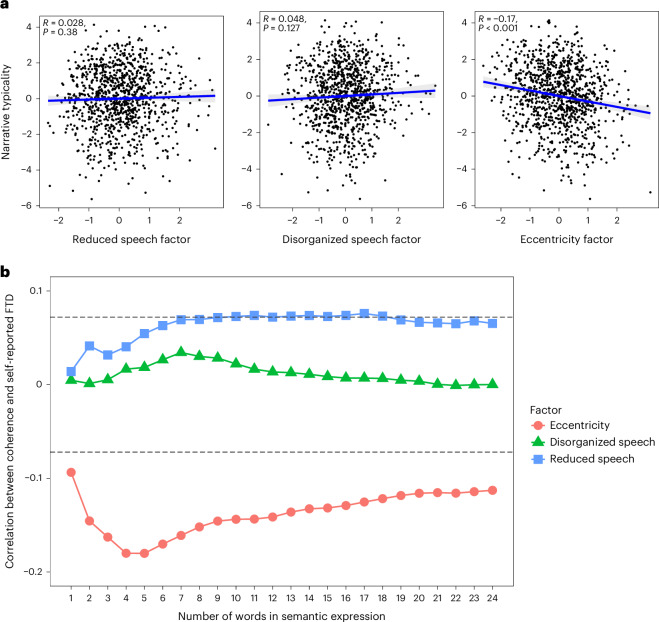
Language atypicality and incoherence in participants with self-reported communication difficulties. **a**,**b**, Correlations between self-reported dimensions of psychopathology (standardized factor scores) subsuming abnormalities in speech and objective atypicality (**a**; depicting the linear regression line and 95% credible interval) and incoherence (**b**) in free narratives. Narrative typicality was measured as the similarity of each narrative to the narratives produced by all other participants in response to a given probe (after downweighting highly common, and thus less informative, words; that is, term frequency–inverse document frequency similarity). Coherence was measured as the inverse of the aggregate minimum amount of distance that each word in one group of words has to move to reach its closet word in the consecutive group of words (word mover’s distance^60^; Methods). These distances were calculated using a pretrained fastText semantic space model (Methods). Both typicality and coherence were standardized and summed across the two narratives for each participant. The horizontal lines in **b** represent the false-discovery-rate-corrected two-tailed significance threshold (corrected across time points and dimensions). *P* values in **a** represent the non-corrected two-tailed significance.

To examine whether self-reported communication difficulties reflect objectively atypical or incoherent use of language, we asked participants to produce narratives in response to two prompts: first, we asked them to relate the story of *Cinderella*; second, we asked them to describe the daily routine of an average person. After standard preprocessing, we measured the typicality of each narrative in terms of its similarity to narratives produced by all other participants to a given prompt. Participants with higher self-reported eccentricity scores produced more atypical narratives (*r* = −0.17, *P* < 0.001), as depicted in Fig. 3a. This relationship could not be explained by demographic variables and verbal working memory (Supplementary Results 1). Consistent with the classic presentation of FTD where ideas drift over time from the original starting ‘point’ (tangentiality), the atypicality shown by people with high eccentricity increased through the unfolding of the narrative (that is, it was more pronounced in the 2nd half of their narratives; interaction *β* = 0.08, s.e. = 0.04, *Z* = 2.03, *P* = 0.042; Supplementary Results 2). Finally, we found no relationship between narrative typicality and the disorganized speech or reduced speech dimensions (all |*r*| < 0.048, *P* values >0.127). Further highlighting the specific role of the eccentricity dimension, narrative atypicality did not correlate with the higher-level transdiagnostic dimensions.

Next, we examined whether narratives produced by people with high eccentricity scores are not only atypical but also less coherent, in the sense of being characterized by reduced semantic similarity between consecutive words or more extended semantic expressions (that is, groups of words of varying size). Our analysis showed loosened associations between semantic expressions exclusive to those with high self-reported eccentricity, particularly for small- to medium-sized semantic expressions (Fig. 3b). Further analysis suggested that this reflects both global and local reduction in semantic coherence, such that semantic content is both globally dispersed and suboptimally organized at the local level (Supplementary Results 3).

Overall, this pattern of findings suggests that the discourse of individuals high in self-reported eccentricity is more atypical, tangential and semantically loosened, portraying a dimensional counterpart of classical FTD. This can be explained by one of several potential mechanisms (Fig. 1) and, in the next section, we consider these mechanisms in detail.

### Mechanisms underlying language atypicality in eccentricity

To examine mechanisms underlying discourse incoherence in eccentricity, we investigated the dynamics through which people generate and regulate associations. FTD has been previously linked to abnormalities in the processing of written linguistic cues^9,33,34^. Thus, to ensure our measure of associative thinking does not depend on the processing of written words, we asked participants (with no color blindness) to produce free associations to colors rather than to words. As expected, and consistent with the free-narrative results (Supplementary Results 4), people with high self-reported eccentricity reported more infrequent (*β* = −0.04, s.e. = 0.009, *Z* = −4.50, *P* < 0.001) and idiosyncratic (that is, those not reported by any other participant; odds ratio = 1.12, *Z* = 4.23, *P* < 0.001) associations. No such effects were found for the disorganized speech or reduced speech dimensions (*P* values ≥0.47). Finally, people with high eccentricity also tended to be slightly slower in starting to write their associations (*β* = 85.16 ms, s.e. = 43.39, *Z* = 1.96, *P* = 0.049).

To arbitrate between possible mechanisms underlying such atypical, slightly delayed associations, we fitted an SMP model, formalizing the interaction between associative thinking and the regulation of thought expression^30^. The SMP model, fitted jointly to the reported associations and reaction times (RTs), builds on the idea that rejecting and replacing an association is costly in terms of time, while also dissociating this effect from other factors affecting RT (for example, general slowness or the extent to which weaker associations are slower to come to mind)^35^.

**Fig. 4:**
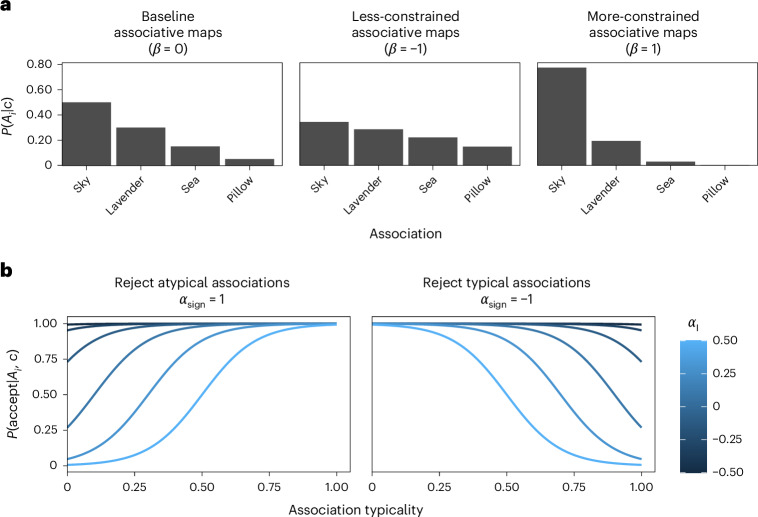
The computational (SMP) model key parameters. **a**,**b**, Illustration of the effects of the three key computational parameters controlling the generation (**a**) and regulation (**b**) of associations in the SMP. When *β* = 0, the probability of generating an association is proportional to how typical this association is (**a**), whereas disorganized thinking was formalized as associative maps that are less constrained by the typicality of associations (*β* < 0). The process of regulating which associations are accepted versus rejected is governed by two parameters (**b**) formalizing the probability of rejecting associations (*α*_I_), and the type (that is, typical/atypical) of rejected associations (*α*_sign_), whereby higher *α*_I_ increases the extent (or probability) of regulation. Association typicality is measured as the proportion of participants reporting the respective association for the given cue.

The model assumes that the probability that a participant samples a certain association is related to its typicality (that is, its relative frequency in the respective color across participants) and a free parameter *β*, which controls the strength of this relationship for a given participant. Specifically, more negative *β* values create a less lopsided distribution in which typical and atypical associations become more similar in strength (Fig. 4a), reflecting less-constrained associative thinking (Fig. 1b). After an association is sampled, it can still be rejected if it is inconsistent with a participant’s goals. Here we assumed that the probability of rejecting an association can either decrease or increase as a function of its typicality, reflecting deliberate creativity (Fig. 1d) or an inhibition of expression of atypical associations (Fig. 1b versus Fig. 1c), respectively. This rejection process is controlled by two parameters, *α*_I_ and *α*_sign_, controlling the strength and the direction of this relationship, respectively (Fig. 4b).

**Extended Data Fig. 2:**
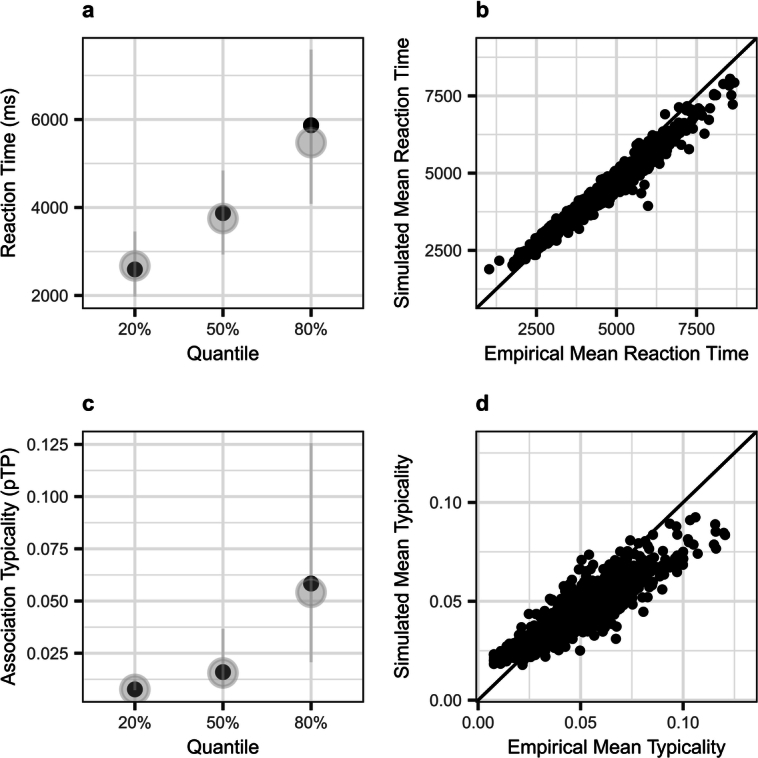
The ability of the semi-Markov process model to explain reaction times and association typicality. The ability of the semi-Markov process model to explain the average distributions of RTs (**a**) and the typicality of associations (**c**), as well as individual differences in mean RT (**b**) and typicality (**d**; scatterplots wherein each dot represents a participant). In Panels **a** and **c**, the black dots represent the respective RT quantiles (calculated for each participant and then averaged), whereas the grey dots and intervals represent average model predictions, and the 2.5% and 97.5% quantiles, calculated across 100 simulations based on best-fitted parameter values.

First, we examined the assumption that participants do not always report the very first association that comes to mind. In support of this, model comparison suggested that models in which people never reject associations perform worse in explaining the data (Supplementary Results 5). The best-fitting model was also able to explain the distributions of speed and typicality of the reported associations at a group level, individual differences in average speed and typicality (Extended Data Fig. 2), and showed satisfactory parameter recoverability (Supplementary Results 5).

Examining individual differences in the key parameters showed that people with high eccentricity have less-constrained associative distributions (*r*_*β*,eccentric_ = −0.12, *P* < 0.001) and are less likely to reject atypical associations (${r}_{{\alpha }_{\rm{I}},\rm{eccentric}}$ = −0.07, *P* = 0.02). Conversely, there was not sufficient evidence of deliberate creativity in eccentricity (${r}_{{\alpha }_{\rm{sign}},\rm{eccentric}}$ = −0.06; *P* = 0.074; Supplementary Results 6). When controlling for rejection probability (*α*_I_), eccentricity remained related to underconstrained associative maps (*r*_(*β*,eccentric).*α*I_ = −0.10; *P* = 0.002). Conversely, eccentricity no longer predicted lower rejection probability when controlling for *β* (*r*_(*α*I,eccentric).*β*_ = −0.03; *P* = 0.359).

**Fig. 5:**
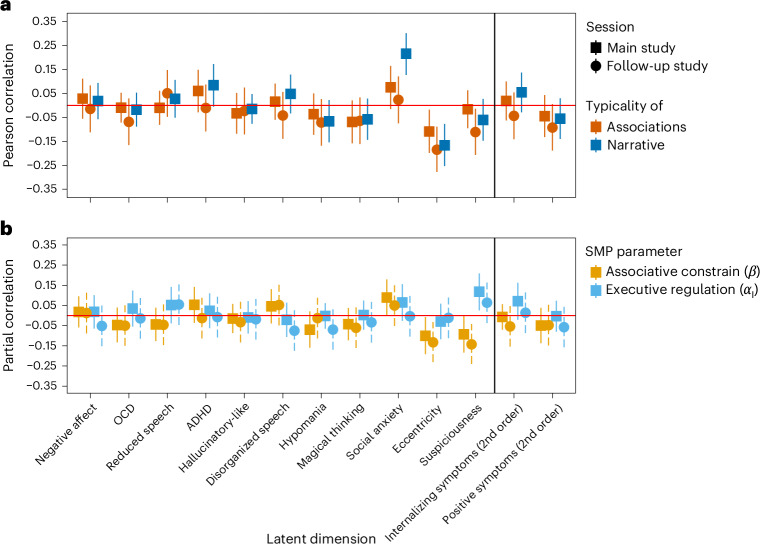
Overt and covert alterations in thought and language across different psychopathological dimensions. **a**,**b**, Effects are presented as correlations between each dimension and the main behavioral measures (**a**) and computational parameters (controlling for each other) (**b**) used in the study. The horizontal line demarcates the two higher-order factors (to the right). Error bars represent confidence intervals (*N*_main study_ = 964, *N*_follow-up_ = 379). The confidence intervals in the main study were corrected for multiple comparisons using the Holm–Bonferroni correction (13 comparisons; no correction was used for the confirmatory follow-up study). Vertical bars represent a null correlation.

**Extended Data Fig. 3:**
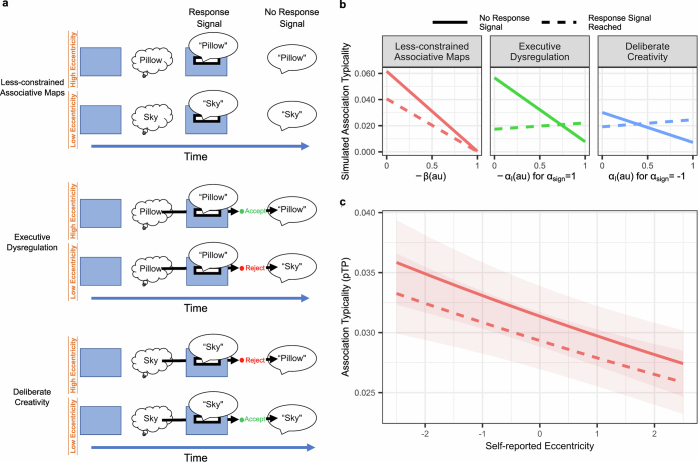
Diminishing executive control by urging quick responses (response signal). Illustrative and formal predictions are depicted in Panels **a** and **b**, respectively, and the empirical results are depicted in Panel **c**. The effects of two of the three potential mechanisms explaining atypical associations vary between trials with no response signal (solid lines in Panels **b** and **c**) and trials where a response signal has been presented (dashed lines in Panels **b** and **c**). The predictions presented in Panel b were derived by allowing only one parameter to vary across participants (The X axis in these plots was min-max normalized; see Supplementary Results 7). Panel c presents the empirical results as GLMM (a Beta regression accounting for the nature of the dependent variable + 95% credible interval), wherein both simple effects are significant (*p*’s < .001), as predicted by less-constrained associative maps.

These results implicate underconstrained associative maps in self-reported eccentricity. Whereas executive dysregulation explains why such underconstrained associations are reported, it falls short as the exclusive explanation for the increased production of atypical associations in these participants. To further establish the key role of underconstrained associative thinking and the stability of our results, we conducted a preregistered follow-up experiment, consisting of a subsample of 401 participants from the original sample (~6 weeks after the main session). In this experiment, executive regulation was experimentally controlled by occasionally presenting a ‘response signal’, urging participants to respond as quickly as possible. This study supported the stability of our results (Fig. 5) and showed that people with high self-reported eccentricity produce more atypical associations whether executive regulation is minimized or not (Extended Data Fig. 3 and Supplementary Results 7).

Finally, the fact that participants viewed some of the colors twice (that is, in the main study and in the follow-up study) allowed us to examine another prediction of less-constrained (and thus more random) associative thinking (Fig. 1b): namely, a reduced probability of repeating the same association to the same color. As expected, people with high eccentricity were less likely to repeat associations for a given color (odds ratio = 0.67, *Z* = −2.64, *P* = 0.008). Together, these findings further support the hypothesis that atypical associations in high eccentricity primarily reflect atypical, less-constrained thinking.

### Evidence for concealed thought disorganization

Our analyses so far focused on psychopathological dimensions that involved self-reported communication difficulties. However, people who score high on other dimensions can show atypical language use but not report it. Furthermore, as noted above, our modeling approach enables capturing covert disorganization in associative thinking. Accordingly, we explored whether other psychiatric dimensions are characterized by either objective alterations in language or covert disorganization in associative thinking.

As shown in Fig. 5a, only elevated eccentricity predicted atypical narratives or associations (Supplementary Results 8). Intriguingly, a ‘suspiciousness’ dimension, capturing a difficulty trusting and confiding in other people (“I tend to keep my feelings to myself”) was associated with covert thought disorganization (that is, low *β*) that had an attenuated linguistic manifestation due to elevated regulation (that is, high *α*_I_; controlling for *β* due to their cross-correlation). As predicted based on this finding, an elevated regulation was also manifest in slower responding (*β* = 199.68 ms, s.e. = 43.67, *Z* = 4.57, *P* < 0.001).

**Extended Data Fig. 4:**
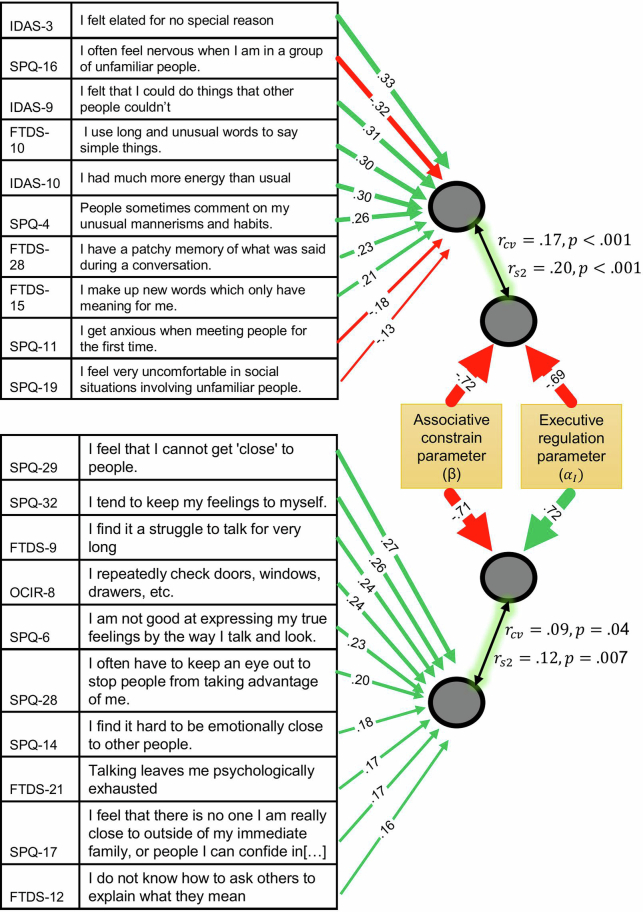
Canonical correlations between questionnaire items and model parameters. Sparse canonical correlation analysis using nested cross-validation, indicating two significant canonical correlations (using a permutation test; see Supplementary Results 9). To enhance interpretability, only the ten items with the highest weights are presented for each latent psychopathological dimension. For the main study cross-validated canonical correlations are presented (r_cv_). The weights fitted based on the main study were then used to calculate the canonical correlations for the follow-up study (r_s2_).

Although the idea that suspiciousness fuels a motivation to conceal atypical associations makes intuitive sense, it may seem incongruent with a clinical dissociation between disorders in the content of thought (that is, persecutory delusions) and disorders in the form of thought (that is, FTD). However, a lack of covariation in symptoms does not preclude a shared computational mechanism. Indeed, the analyses above define psychopathological dimensions solely based on symptom covariation, whereas an alternative approach involves incorporating knowledge of mechanisms into the definition of the transdiagnostic dimensions themselves. For this purpose, we used sparse canonical correlation analysis to define latent dimensions of psychopathology based on their purported underlying computational mechanisms. The results of this analysis supported disorganized thinking as a core mechanism that interacts with either under- or over-regulation to produce distinct behavioral profiles (Extended Data Fig. 4 and Supplementary Results 9). Thus, whereas increased self-reported eccentricity in speech and behavior (but also, interestingly, some hypomanic symptoms and reduced social anxiety) predicted less-constrained associative maps and executive dysregulation, difficulties trusting and confiding in other people (but also a reported reduction in speech) predicted less-constrained associative maps with increased regulation of speech.

## Discussion

Humans do not express all that comes to mind and often use executive processes to inhibit irrelevant ideas^36^. Yet, psychiatric nomenclature often equates incoherent communication with disorganized thought, as implied by the ascription of FTD. The latter’s strong diagnostic emphasis on overt speech risks overlooking covert forms of thought disorganization and misconceptualizing underlying cognitive processes. Indeed, whereas alterations in language have been suggested as a biomarker for schizophrenia^24,37–39^, the generality and specificity of this relationship are debated^11,12,15,16,25,40,41^. These questions provided the motivation for us to examine language and covert thought across a range of latent dimensions of psychopathology.

Our findings suggest a core mechanism underlying FTD is more widespread than commonly assumed, while also showing specificity to particular dimensions of psychopathology. In particular, we found evidence for disorganized thinking as a core mechanism in self-reported eccentricity and suspiciousness. Critically, whereas self-reported eccentricity was characterized by atypicality in overt language, reflecting a secondary executive impairment, thought disorganization remained covert in those with high suspiciousness. These results extend previous findings highlighting the involvement of both associative and executive impairments in clinical FTD^5,6^, offering new insights into how these processes interact differently in different dimensions of psychopathology.

The aforementioned specificity is highlighted by an absence of evidence for alterations in associative thinking or discourse in other dimensions reflecting psychotic-like symptoms (for example, hallucinations), self-reported tendencies for long-winded speech, or non-psychotic psychopathologies associated with self-reported disorganization or unpredictability of thought (that is, obsessive–compulsive disorder and ADHD^15–18^). These results are notable given recently expanding evidence that many cognitive, computational and linguistic markers are widely transdiagnostic^19,22,42^.

Notably, the psychiatric dimensions that predicted an alteration in language or thought are all of an interpersonal nature (Supplementary Results 10), echoing previous research on social functioning impairments in FTD^43–46^. The latter has been suggested to reflect a vicious cycle during development: atypical communication is met by uneasiness from peers, leading to isolation and reduced social feedback, and ultimately impairing social cognition and semantic alignment^47^. Our results extend on this by suggesting distinct interpersonal symptoms linked to unique alterations in language and thought. Speculatively, this variation might reflect different coping mechanisms with respect to interpersonal difficulties. For example, a person might accept ‘being different’ (as in self-reported eccentricity), feel compelled to conform and avoid being different (as in social anxiety) or lose trust and conceal their internal world (as in suspiciousness).

Our study has several limitations. In accordance with the hypothesized socially situated nature of FTD, our measurements of language and thought (which involve typing in non-interacting online participants) disregard many important linguistic and social aspects of natural (and clinical) discourse. Nonetheless, whereas our study focused on subtle alterations in language rather than clinical manifestations of FTD, our dimensional approach enables a fine-grained computational phenotyping that can help disentangle the interacting mechanisms of more complex clinical phenomena.

In examining the role of executive dysregulation, we focused on how people regulate the expression of thoughts, rather than how they directly control which thoughts are generated^30^. Indeed, whereas our analysis focused on spontaneous semantic and executive processes, future studies measuring these processes under instructions encouraging either typical or creative output could help delineate individual differences in various control processes, and dissociate pathological atypicality from creative thinking^31^.

Overall, our findings provide for a mechanistically informed account of alterations in language and thought in psychopathology that goes beyond traditional diagnostic and symptom-based boundaries yet allows for notable specificity. The results also suggest that thought and language are socially situated, and that a disruption in the form of thought is intimately intertwined with a compromise of interpersonal development and social function. This underlines a need to broaden the study of thought dynamics to include tasks that incorporate interpersonal exchange, thereby paving the way for a deeper perspective on how thought and communication evolve within a social context.

## Methods

### Participants

The study has been approved by the University College London research ethics committee (number 16639/001). All participants provided written informed consent and were compensated for their time. A total of 1,100 adult, UK-based participants were recruited via the Prolific Academic Internet platform. Participants failing ≥1 of 5 attention check questions (Supplementary Methods 1) were excluded from further analysis (*N* = 100). The final sample included 625 women and 360 men who were 38.22 (s.d. = 13.01) years old on average. Participants varied in the highest level of education acquired (high school for 230 participants, level 4+ qualification for 160 participants, a bachelor’s degree for 381 participants, and a master’s degree or higher for 211 participants). Participants were only allowed to participate if they reported being raised monolingual with English as their first language. The majority of participants reported receiving their primary and secondary education in an English-speaking country (97.9% and 98.0%, respectively). Note that 5 participants did not properly complete the color association task and an additional 31 participants had less than 10 valid responses in this task; therefore, these participants were excluded from modeling analysis (Supplementary Methods 1).

A total of 401 participants (60.59% women, mean age = 41.86 years, s.d. = 12.09 years) were invited to participate in a preregistered follow-up study (https://aspredicted.org/tmzc-xyft.pdf) designed to test the stability of the modeling results and examine unregulated associative thinking. This sample size was determined based on a preregistered power analysis. Finally, to screen for objective (rather than only self-reported) color blindness, the follow-up study started with 15 plates from the Ishihara color blindness test, and only participants scoring above 90% were allowed to participate. The method and results of this study are reported in detail in Supplementary Results 7.

### Self-report psychiatric questionnaires and factor analysis procedure

Participants were administered seven psychiatric questionnaires with potential relevance to thought disorganization and communication difficulties. These included questionnaires measuring schizotypy^48^, self-reported FTD^49^, hallucinatory experiences^50^, hypomania symptoms^51^, ADHD symptoms^52^, obsessive–compulsive symptoms^53^, and general depression and anxiety^54^.

To infer the latent transdiagnostic dimensions explaining the correlations among the items included in these questionnaires, we used exploratory factor analysis while accounting for the ordinal nature of these items. First, to estimate the most likely number of latent factors, we used the highly recommended parallel analysis method^55^ (using minimum rank factor analysis with polychoric correlations as an extraction method^56^). Then, the factor structure and factor scores were estimated in Mplus (v.7), using the unweighted mean- and variance-adjusted least-squares estimator (ULSMV; suitable for ordinal items) and oblimin rotation, designed to reduce cross-loadings.

We note that our factor solution differs considerably from previous transdiagnostic studies, producing three broader factors^20,32^. The reasons for this include the different questionnaires and the different factor retention method we chose based on its better performance in recovering the data-generating number of factors^55^. Yet, to examine whether broader factors may more parsimoniously explain alterations in thought and language, we conducted a higher-order factor analysis by submitting the factor scores derived from the procedure above to an additional factor analysis (Extended Data Fig. 1).

### Free-narrative task and NLP methods

To measure a range of indicators of atypicality and incoherence in natural language use, we asked participants to write free narratives describing two topics in as much detail as possible. Our choice to collect written rather than oral narratives was motivated by the inherent difficulties in recording and transcribing the responses in a large, internet-based sample. We encouraged participants to imagine themselves ‘trying to explain the answer to someone’ while mimicking the critical feature of oral communication where words cannot be redacted once uttered. Specifically, each time a participant pressed the space bar or the enter keys, the previous word disappeared. Participants were first trained on this unconventional way of typing by asking them to copy the task instructions. They were then asked to ‘describe in detail the story of *Cinderella*’ and ‘describe in detail what steps are usually involved in people’s daily routine’ (in random order). Participants were required to write at least 100 words (2-letter words were not counted) per narrative to proceed.

Participants’ narratives were segmented into words and preprocessed according to standard procedures using the NLTK python library. Preprocessing included the removal of non-words/misspelled words (4.97%), digits (0.2%), classical ‘stop words’ (that is, determiners, coordinating conjunctions and propositions; 49.41%), single letters (0.08%) and other non-informative words that were highly common in participants’ narratives (for example, ‘usually’, ‘made’ and ‘thing’; 2.3%). We also removed words typed immediately before or after the participant navigated away from the experiment’s screen, as this might indicate an external distraction that could artificially reduce coherence (0.03%). Finally, we applied basic lemmatization to convert words to their dictionary form (for example, ‘chores’ to ‘chore’ and ‘going’ to ‘go’).

The preprocessed narratives were then submitted to two types of NLP analyses. First, to measure narrative typicality, we calculated the similarity between each participant’s narrative and the respective narratives of all other participants, underweighting words repeating across all narratives (using term frequency–inverse document frequency weighting; Supplementary Methods 2). This measure of narrative typicality has shown good split-half reliability (*r* = 0.62), with a medium-sized correlation between the two narratives (*r* = 0.41), suggesting that although most of the variance in typicality is narrative specific, a substantial portion of the variance is explained by a shared latent factor. The correlations with psychiatric dimensions were thus calculated based on an aggregate narrative typicality score.

Second, to measure the internal coherence of the narratives, we examined the semantic similarity between consecutive words (or groups of words) using several popular embedding models, which represent words as vectors in a multidimensional space and thus allow quantifying similarity using a measure of cosine similarity^25^. We examined three popular pretrained embedding models, namely, Word2vec (a 300-dimension space trained on Google news^57^), Global Vectors for Semantic Representation (GloVe; a 300-dimension space trained on Wikipedia and the Gigaword corpus^58^) and fastText (a 300-dimension space trained on Common Crawl and Wikipedia^59^). Importantly, testing three different models, each trained on a different corpus, ensures that our results cannot be explained by a specific model or corpus.

We compared two methods for measuring narrative coherence at higher levels of representation (that is, how related groups of words are). First, the word-level vectors were aggregated across varying levels (that is, *N* content words) and the cosine similarities between consecutive aggregated vectors were computed. Second, we quantified the distance between groups of *N* words as the ‘word mover’s distance’^60^, which quantifies the aggregate minimum amount of distance that each word in one group has to ‘move’ to reach its closet word in the second group. Both procedures were repeated and averaged across different starting points (for example, for the phrase ‘*Cinderella* was a *beautiful girl* with a *wicked mother*’ and a window size of two, we calculated the similarity between Cinderella + beautiful and girl + wicked, and the similarity between beautiful + girl and wicked + mother, and then averaged across starting points).

Figure 3b presents the coherence results using word mover’s distance based on fastText embeddings, because this metric and this model produced an optimal combination of high shared variance between the two narratives and relatively low correlation with the typicality measure specified above (Supplementary Methods 2). Yet, consistent results were found for other embedding models and metrics (Supplementary Methods 2).

We note that tangentiality, a common FTD symptom defined as the tendency of narratives to drift away from their original topic, has been previously measured based on how semantic distance from an initial prompt increases over the course of a narrative (that is, slope)^61^. However, as we found a negligible correlation between such slope measures across the two narratives (indicating negligible shared variance), we do not use this measure here. Similarly, whereas narrative organization has been previously operationalized using content-based measures aiming to delineate a common structure^62^, such measures also showed too little shared variance among the two stories elicited here (*r* = −0.003). More generally, our focus on measures of organization and ‘form’ led us to forgo other, potentially informative NLP measures focusing on content (for example, counting specific, psychologically informative words)^63^.

### Color association task

On each trial, a color is presented across the entire screen and participants are asked to provide an association to that color. Colors that do not correspond with any real-world objects might evoke no association at all. Thus, instead of sampling colors randomly, we sampled 148 relatively light (light > 0.2) and saturated (saturation > 0.1) colors from a large dataset of fruit images (Food-11 dataset). As colors are often similar to each other, presenting participants with a large number of colors might evoke many repeated associations. Thus, each participant was presented with a random subset of 20 colors. Furthermore, as even natural colors might not evoke any associations, we allowed participants to skip up to 50% of the remaining trials. However, this option was rarely used in practice (0.96 trials on average; 75% of participants skipped only 1 trial or less). In the follow-up study, all participants were presented with 50 colors, preselected to remove colors to which participants struggled the most to find an association and minimize overlap among colors. Also note that whereas in the main study participants were asked to provide a ‘concrete noun’, no such limitation was posed in the follow-up study to ensure generalizability.

In the main study, RTs were measured based on the timing of the first typed letter. As this measure might be contaminated by variability in typing speed, in the follow-up study we opted to dissociate the time it takes to think of an association from the time it takes to write it by asking participants to first press the space bar as soon as an association came to mind and only then type in their association and press enter, while using a dedicated procedure nudging participants to avoid premature presses^30,35^.

The primary dependent variable in the color association task was the typicality of an association. After removing non-word or misspelled responses (6.84% in the main study and 7.82% in the follow-up; spelling was tested using the Hunspell R package), color names (1.50% and 2.22%, respectively) and single-letter responses (0.27% and 0.02%, respectively), we used basic lemmatization to convert all words to their basic dictionary form (Textstem R package). Then, association typicality was measured as the proportion of participants endorsing that association. To account for this variable’s bounded, non-normal nature, we used generalized linear mixed effects models (GLMMs)^64^ in which typicality was assumed to be distributed in a beta distribution, with a logit link function and random intercepts for participants and color. This analysis was augmented by an analysis of idiosyncrasy, where we used a logistic GLMM to determine which psychiatric dimensions predicted the report of an association that was not reported by any other participant. Indeed, whereas rare, non-idiosyncratic responses could be interpreted as creative (and appropriate^65^), idiosyncratic associations are often considered completely unrelated^65,66^. Analyses predicting RTs were based on a GLMM with a gamma-distributed response variable (with an identity link function). In multiple regression models, all predictors were standardized.

### SMP model

The SMP model is designed to formalize the cognitive process through which candidate associations are generated and regulated. As shown in equation (1), the probability that a specific association *A*_*i*_ is generated in response to color *c* is assumed to be a function of the typicality of this association (pTP_*i*,*c*_) and a free parameter, *β* (fitted at the participant level, here denoted by *s*), controlling the extent to which typical associations are more likely to be generated:

1 $$P{({A}_{i}|c)}_{s}=\frac{{\rm{pTP}}_{i,c}^{\exp ({\beta }_{s})}}{{\sum }_{i=1}^{I}{{\rm{pTP}}_{i,c}^{\exp ({\beta }_{s})}}}$$

After an association is generated, it can be either rejected or accepted and reported with a probability that is either monotonically or inversely related to its typicality:

2 $$P({\rm{accept}}|{A}_{i},c)_{s}=\left\{\begin{array}{cc}\frac{1}{1+\exp [-10({\rm{MX}}({\rm{pTP}}_{i,c})-{\alpha }_{{\rm{I}}_{\rm{s}}})]} & {\rm{if}}\,{\alpha }_{{\rm{sign}}_{\rm{s}}}=1\\ \frac{1}{1+\exp [-10({\rm{MX}}(1-{\rm{pTP}}_{i,c})-{\alpha }_{{\rm{I}}_{\rm{s}}})]} & {\rm{if}}\,{\alpha }_{{\rm{sign}}_{\rm{s}}}=-1\end{array}\right.$$

where *α*_I_ and *α*_sign_ are free parameters and MX is a min–max transformation:

3 $${\rm{MX}}({\rm{pTP}}_{i,c})=\frac{{\rm{pTP}}_{i,c}-\,\min ({\rm{pTP}}_{i,c})}{\max ({\rm{pTP}}_{i,c})-\,\min ({\rm{pTP}}_{i,c})}$$

This type of normalization was chosen because it minimizes the effect of cue entropy (for example, the number of different associations given across participants) on rejection probabilities. Finally, the slope of 10 in equation (2) was chosen to allow for a relatively meaningful association with pTP; the parameters of a model where this slope was a free parameter were not recoverable.

On the basis of previous findings^35^, we assume that stronger associations take less time to generate than weaker ones. That is, the time it takes to generate each candidate association is a function of its probability (for a given participant) and free parameters modulating this relationship. The overall RT per reported association is the sum of the time it takes to generate all candidate associations until an association is accepted, plus the latency of all other processes that are not directly related to the specific associations, such as color-encoding time and typing speed (henceforth non-decision time)^67^. This sum is formulated using an SMP representing the stochastic evolution of a random number of time-varying states (that is, associations)^68^.

Specifically, the time it takes to generate each candidate association is drawn from a gamma distribution. As in a previous application^30^, the mean of this generation-time distribution was positively linked with the surprisal (−log[*P*(*A*_*i*_|*c*)_*s*_]) of an association, such that weaker associations are, generally, slower:

4 $${\mu }_{i(c,s)}=\exp ({\rm{S}}{\rm{\mu}}_{s}) (-\,\log [P({A}_{i}|{c})_{s}])$$

This functional form assumes a mean thinking time of 0 in the hypothetical case of a cue with a single association, whereas Sμ is a free parameter controlling the slope of the function linking an association’s probability to its mean generation time. Following a previous application of the SMP, the standard deviation of this generation-time distribution was parameterized as a function of its mean^30^:

5 $${\sigma }_{i(c,s)}=\lambda_s {\mu }_{i(c,s)}$$

where *λ* is a free parameter. This parameterization explained the data better than several alternatives (for example, using an exponential distribution and using a nonlinear function linking surprisal to mean generation time) as shown in Supplementary Results 5.

Together, equations (1)–(5) are used to specify two matrices: (1) a matrix defining the probabilities of transitioning between states (for example, from the color, or a rejected association, to the next candidate association), and (2) a matrix of parameters determining how long it takes to complete each transition. These matrices are used to derive the joint probability density function of the reported association, and the time it takes to choose it, using a Laplace transformation^68^ (note that use of a gamma distribution to model sampling times was motivated by its relatively simple Laplace transformation).

As noted above, RTs reflect not only the time it takes to choose an association but also additional non-decision time (for example, motor speed and cue encoding). Non-decision time in many evidence accumulation models is simplified to a constant value to retain tractability. Conversely, here we can exploit the fact that the SMP uses a Laplace transformation to account for the convolution of multiple generation-time distributions (in the case of rejections) to also allow non-decision time to vary across trials while retaining tractability. As we explain below, this is also particularly important when parameters are fitted hierarchically (such that participant-level non-decision time is derived from a group-level parameter) and participants vary dramatically in their minimum RT. Thus, non-decision time was assumed to be distributed in a uniform distribution with a range *τ*_*r*_ and a minimum *τ*_0_. Overall, the SMP used here had seven free parameters.

Participant-level parameters were fitted using an iterative hierarchical expectation-maximization procedure^69^, designed to compensate for the relatively small number of trials from each participant by using empirical group-level priors to constrain participant-level parameters. First, for each participant, we sampled 1,000 different random settings of parameters from non-informative predefined group-level prior distributions (Supplementary Methods 3). Then, for each participant and parameter setting, we computed the joint likelihood of their associations and RTs for all trials. These likelihoods were then used as importance weights to refit the parameters of the group-level prior distributions. These steps were repeated iteratively until model evidence (integrated Bayesian information criteria)^69^ ceased to decrease. In practice, this required 9–15 iterations, depending on the parameterization. Note that because the number of parameters is relatively large (that is, 7), 1,000 possible combinations are not enough to explore the entire parameter space. This relatively small number was obligated by the fact that estimating the likelihood for each trial takes a relatively long time in the SMP^30^. However, our large sample and the fact that different parameter settings were sampled for each participant ensured an extensive exploration of the space of possible parameter combinations (that is, 964,000 in total in each iteration). Finally, to derive the best-fitting parameters for each individual participant, we sampled a single sample of 10,000 random settings of parameters from the final group-level empirical priors and computed a weighted mean, based on the participant-level likelihood for each setting. Note that in contrast to the iterative procedure described above, here we tested the same parameter settings across participants to ensure that individual differences in best-fitted parameters are not affected by random variation in the tested parameter settings.

### Reporting summary

Further information on research design is available in the Nature Portfolio Reporting Summary linked to this article.

## Supplementary information

Supplementary Information Supplementary Figs. 1–9, Tables 1–8, Results 1–10 and Methods 1–3.

Reporting Summary

## Data Availability

Data used for analysis, including questionnaire scores, NLP measures derived from participants’ narratives and data from the color association task, can be found at https://osf.io/58k7y/. Per study protocol and the consent form, participant’s actual associations and narratives are available upon request from the corresponding author.

## Code availability

All scripts used for analysis and modeling are available at https://osf.io/58k7y/.

## References

[1] Vanaken, L. & Hermans, D. Be coherent and become heard: the multidimensional impact of narrative coherence on listeners’ social responses. *Mem. Cognit.* **49**, 276–292 (2021). 32901416 10.3758/s13421-020-01092-8 PMC7886714

[2] Cavelti, M., Homan, P. & Vauth, R. The impact of thought disorder on therapeutic alliance and personal recovery in schizophrenia and schizoaffective disorder: an exploratory study. *Psychiatry Res.* **239**, 92–98 (2016). 27137967 10.1016/j.psychres.2016.02.070

[3] Roche, E., Creed, L., MacMahon, D., Brennan, D. & Clarke, M. The epidemiology and associated phenomenology of formal thought disorder: a systematic review. *Schizophr. Bull.* **41**, 951–962 (2015). 25180313 10.1093/schbul/sbu129 PMC4466171

[4] Hart, M. & Lewine, R. R. J. Rethinking thought disorder. *Schizophr. Bull.* **43**, 514–522 (2017). 28204762 10.1093/schbul/sbx003 PMC5464106

[5] Kerns, J. G. & Berenbaum, H. Cognitive impairments associated with formal thought disorder in people with schizophrenia. *J. Abnorm. Psychol.* **111**, 211–224 (2002). 12003444

[6] Barrera, A., McKenna, P. J. & Berrios, G. E. Formal thought disorder in schizophrenia: an executive or a semantic deficit? *Psychol. Med.* **35**, 121–132 (2005). 15842035 10.1017/s003329170400279x

[7] Bora, E., Yalincetin, B., Akdede, B. B. & Alptekin, K. Neurocognitive and linguistic correlates of positive and negative formal thought disorder: a meta-analysis. *Schizophr. Res.* **209**, 2–11 (2019). 31153670 10.1016/j.schres.2019.05.025

[8] Sumner, P. J., Carruthers, S. P. & Rossell, S. L. Examining self-reported thought disorder: continuous variation, convergence with schizotypy, and cognitive correlates. *Psychiatry Res.* **289**, 112943 (2020). 32417592 10.1016/j.psychres.2020.112943

[9] Tan, E. J. & Rossell, S. L. Language comprehension and neurocognition independently and concurrently contribute to formal thought disorder severity in schizophrenia. *Schizophr. Res.* **204**, 133–137 (2019). 30126817 10.1016/j.schres.2018.08.019

[10] Doughty, O. J. & Done, D. J. Is semantic memory impaired in schizophrenia? A systematic review and meta-analysis of 91 studies. *Cogn. Neuropsychiatry* **14**, 473–509 (2009). 19894144 10.1080/13546800903073291

[11] Kircher, T. et al. A rating scale for the assessment of objective and subjective formal thought and language disorder (TALD). *Schizophr. Res.* **160**, 216–221 (2014). 25458572 10.1016/j.schres.2014.10.024

[12] Kircher, T., Bröhl, H., Meier, F. & Engelen, J. Formal thought disorders: from phenomenology to neurobiology. *Lancet Psychiatry* **5**, 515–526 (2018). 29678679 10.1016/S2215-0366(18)30059-2

[13] Palmier-Claus, J. et al. Cognitive behavioural therapy for thought disorder in psychosis. *Psychosis* **9**, 347–357 (2017).

[14] Barron, F. Controllable oddness as a resource in creativity. *Psychol. Inq.* **4**, 182–184 (1993).

[15] Fradkin, I., Eitam, B., Strauss, A. Y. & Huppert, J. D. Thoughts as unexpected intruders: context, obsessive–compulsive symptoms, and the sense of agency over thoughts. *Clin. Psychol. Sci.* **7**, 162–180 (2019).

[16] Fradkin, I. & Huppert, J. D. When our train of thought goes off track: the different facets of out-of-context thoughts in obsessive compulsive disorder. *J. Obsessive Compuls. Relat. Disord.* **18**, 31–39 (2018).

[17] Bozhilova, N. S., Michelini, G., Kuntsi, J. & Asherson, P. Mind wandering perspective on attention-deficit/hyperactivity disorder. *Neurosci. Biobehav. Rev.* **92**, 464–476 (2018). 30036553 10.1016/j.neubiorev.2018.07.010 PMC6525148

[18] Martz, E., Weibel, S. & Weiner, L. An overactive mind: investigating racing thoughts in ADHD, hypomania and comorbid ADHD and bipolar disorder via verbal fluency tasks. *J. Affect. Disord.* **300**, 226–234 (2022). 34958814 10.1016/j.jad.2021.12.060

[19] Hansen, L. et al. Speech- and text-based classification of neuropsychiatric conditions in a multidiagnostic setting. *Nat. Mental Health*10.1038/s44220-023-00152-7 (2023).37250466

[20] Rouault, M., Seow, T., Gillan, C. M. & Fleming, S. M. Psychiatric symptom dimensions are associated with dissociable shifts in metacognition but not task performance. *Biol. Psychiatry* **84**, 443–451 (2018). 29458997 10.1016/j.biopsych.2017.12.017 PMC6117452

[21] Dubois, M. & Hauser, T. U. Value-free random exploration is linked to impulsivity. *Nat. Commun.* **13**, 4542 (2022). 35927257 10.1038/s41467-022-31918-9 PMC9352791

[22] Wise, T., Robinson, O. J. & Gillan, C. M. Identifying transdiagnostic mechanisms in mental health using computational factor modeling. *Biol. Psychiatry* **93**, 690–703 (2023). 36725393 10.1016/j.biopsych.2022.09.034 PMC10017264

[23] Elvevåg, B. et al. Thoughts about disordered thinking: measuring and quantifying the laws of order and disorder. *Schizophr. Bull.* **43**, 509–513 (2017). 28402507 10.1093/schbul/sbx040 PMC5464160

[24] Corcoran, C. M. et al. Prediction of psychosis across protocols and risk cohorts using automated language analysis. *World Psychiatry* **17**, 67–75 (2018). 29352548 10.1002/wps.20491 PMC5775133

[25] de Boer, J. N. et al. Clinical use of semantic space models in psychiatry and neurology: a systematic review and meta-analysis. *Neurosci. Biobehav. Rev.* **93**, 85–92 (2018). 29890179 10.1016/j.neubiorev.2018.06.008

[26] Voppel, A. E., de Boer, J. N., Brederoo, S. G., Schnack, H. G. & Sommer, I. Quantified language connectedness in schizophrenia-spectrum disorders. *Psychiatry Res.* **304**, 114130 (2021). 34332431 10.1016/j.psychres.2021.114130

[27] Lundin, N. B., Cowan, H. R., Singh, D. K. & Moe, A. M. Lower cohesion and altered first-person pronoun usage in the spoken life narratives of individuals with schizophrenia. *Schizophr. Res.* **259**, 140–149 (2023). 37127466 10.1016/j.schres.2023.04.001 PMC10524354

[28] Tang, S. X. et al. Natural language processing methods are sensitive to sub-clinical linguistic differences in schizophrenia spectrum disorders. *NPJ Schizophr.* **7**, 25 (2021). 33990615 10.1038/s41537-021-00154-3 PMC8121795

[29] Minor, K. S., Willits, J. A., Marggraf, M. P., Jones, M. N. & Lysaker, P. H. Measuring disorganized speech in schizophrenia: automated analysis explains variance in cognitive deficits beyond clinician-rated scales. *Psychol. Med.* **49**, 440–448 (2019). 29692287 10.1017/S0033291718001046

[30] Fradkin, I. & Eldar, E. If you don’t let it in, you don’t have to get it out: thought preemption as a method to control unwanted thoughts. *PLoS Comput. Biol.* **18**, e1010285 (2022). 35834438 10.1371/journal.pcbi.1010285 PMC9282588

[31] Prabhakaran, R., Green, A. E. & Gray, J. R. Thin slices of creativity: using single-word utterances to assess creative cognition. *Behav. Res. Methods* **46**, 641–659 (2014). 24163211 10.3758/s13428-013-0401-7 PMC4105589

[32] Wise, T. & Dolan, R. J. Associations between aversive learning processes and transdiagnostic psychiatric symptoms in a general population sample. *Nat. Commun.* **11**, 4179 (2020). 32826918 10.1038/s41467-020-17977-w PMC7443146

[33] Wang, K., Cheung, E. F. C., Gong, Q. & Chan, R. C. K. Semantic processing disturbance in patients with schizophrenia: a meta-analysis of the N400 component. *PLoS ONE* **6**, e25435 (2011). 22022395 10.1371/journal.pone.0025435 PMC3192062

[34] Prévost, M. et al. Schizotypal traits and N400 in healthy subjects. *Psychophysiology* **47**, 1047–1056 (2010). 20456656 10.1111/j.1469-8986.2010.01016.x

[35] Fradkin, I. & Eldar, E. Accumulating evidence for myriad alternatives: modeling the generation of free association. *Psychol. Rev.* **130**, 1492–1520 (2023). 36190752 10.1037/rev0000397 PMC10159868

[36] Ye, Z. & Zhou, X. Executive control in language processing. *Neurosci. Biobehav. Rev.* **33**, 1168–1177 (2009). 19747595 10.1016/j.neubiorev.2009.03.003

[37] Bedi, G. et al. Automated analysis of free speech predicts psychosis onset in high-risk youths. *NPJ Schizophr.* **1**, 15030 (2015). 27336038 10.1038/npjschz.2015.30 PMC4849456

[38] Rezaii, N., Walker, E. & Wolff, P. A machine learning approach to predicting psychosis using semantic density and latent content analysis. *NPJ Schizophr.* **5**, 9 (2019). 31197184 10.1038/s41537-019-0077-9 PMC6565626

[39] de Boer, J. N., Brederoo, S. G., Voppel, A. E. & Sommer, I. E. C. Anomalies in language as a biomarker for schizophrenia. *Curr. Opin. Psychiatry* **33**, 212–218 (2020). 32049766 10.1097/YCO.0000000000000595

[40] Fradkin, I., Eitam, B., Strauss, A. Y. & Huppert, J. D. How can an overlapping mechanism lead to distinct pathology? The case of psychosis and obsessive compulsive disorder. *Clin. Psychol. Sci.* **7**, 409–410 (2019).

[41] Cohen, A. S., Auster, T., Callaway, D., MacAulay, R. K. & Minor, K. S. Neurocognitive underpinnings of language disorder: contrasting schizophrenia and mood disorders. *J. Exp. Psychopathol.* **5**, 492–502 (2014).

[42] Dalgleish, T., Black, M., Johnston, D. & Bevan, A. Transdiagnostic approaches to mental health problems: current status and future directions. *J. Consult. Clin. Psychol.* **88**, 179–195 (2020). 32068421 10.1037/ccp0000482 PMC7027356

[43] de Sousa, P., Sellwood, W., Eldridge, A. & Bentall, R. P. The role of social isolation and social cognition in thought disorder. *Psychiatry Res.* **269**, 56–63 (2018). 30145302 10.1016/j.psychres.2018.08.048

[44] de Sousa, P., Spray, A., Sellwood, W. & Bentall, R. P. ‘No man is an island’. Testing the specific role of social isolation in formal thought disorder. *Psychiatry Res.* **230**, 304–313 (2015). 26384574 10.1016/j.psychres.2015.09.010

[45] Marggraf, M. P. et al. Speech production and disorganization in schizotypy: investigating the role of cognitive and affective systems. *J. Psychiatr. Res.* **114**, 11–16 (2019). 30991167 10.1016/j.jpsychires.2019.03.023

[46] Palaniyappan, L. More than a biomarker: could language be a biosocial marker of psychosis? *NPJ Schizophr.* **7**, 42 (2021). 34465778 10.1038/s41537-021-00172-1 PMC8408150

[47] Debbané, M. & Barrantes-Vidal, N. Schizotypy from a developmental perspective. *Schizophr. Bull.* **41**, S386–S395 (2015). 25548385 10.1093/schbul/sbu175 PMC4373627

[48] Cohen, A. S., Matthews, R. A., Najolia, G. M. & Brown, L. A. Toward a more psychometrically sound brief measure of schizotypal traits: introducing the SPQ-Brief Revised. *J. Pers. Disord.* **24**, 516–537 (2010). 20695810 10.1521/pedi.2010.24.4.516

[49] Barrera, A., McKenna, P. J. & Berrios, G. E. Two new scales of formal thought disorder in schizophrenia. *Psychiatry Res.* **157**, 225–234 (2008). 17997165 10.1016/j.psychres.2006.09.017

[50] Waters, F. A. V., Badcock, J. C. & Maybery, M. T. Revision of the factor structure of the Launay–Slade Hallucination Scale (LSHS-R). *Pers. Individ. Dif.* **35**, 1351–1357 (2003).

[51] Watson, D. et al. Development and validation of new anxiety and bipolar symptom scales for an expanded version of the IDAS (the IDAS-II). *Assessment* **19**, 399–420 (2012). 22822173 10.1177/1073191112449857

[52] Kessler, R. C. et al. The World Health Organization Adult ADHD Self-Report Scale (ASRS): a short screening scale for use in the general population. *Psychol. Med.* **35**, 245–256 (2005). 15841682 10.1017/s0033291704002892

[53] Foa, E. B. et al. The Obsessive–Complusive Inventory: development and validation of a short version. *Psychol. Assess.* **14**, 485–495 (2002). 12501574

[54] Henry, J. D. & Crawford, J. R. The short-form version of the Depression Anxiety Stress Scales (DASS-21): construct validity and normative data in a large non-clinical sample. *Br. J. Clin. Psychol.* **44**, 227–239 (2005). 16004657 10.1348/014466505X29657

[55] Auerswald, M. & Moshagen, M. How to determine the number of factors to retain in exploratory factor analysis: a comparison of extraction methods under realistic conditions. *Psychol. Methods* **24**, 468–491 (2019). 30667242 10.1037/met0000200

[56] Timmerman, M. E. & Lorenzo-Seva, U. Dimensionality assessment of ordered polytomous items with parallel analysis. *Psychol. Methods* **16**, 209–220 (2011). 21500916 10.1037/a0023353

[57] Mikolov, T., Chen, K., Corrado, G. & Dean, J. Efficient estimation of word representations in vector space. Preprint at https://arxiv.org/abs/1301.3781 (2013).

[58] Pennington, J., Socher, R. & Manning, C. Glove: global vectors for word representation. In *Proc. 2014 Conference on Empirical Methods in Natural Language Processing (EMNLP)* (eds Moschitti, A. et al.) 1532–1543 (Association for Computational Linguistics, 2014); 10.3115/v1/D14-1162

[59] Bojanowski, P., Grave, E., Joulin, A. & Mikolov, T. Enriching word vectors with subword information. *Trans. Assoc. Comput. Linguist.* **5**, 135–146 (2017).

[60] Kusner, M., Sun, Y., Kolkin, N. & Weinberger, K. From word embeddings to document distances. In *Proc. 32nd International Conference on Machine Learning* Vol. 37, 957–966 (PMLR, 2015).

[61] Elvevåg, B., Foltz, P. W., Weinberger, D. R. & Goldberg, T. E. Quantifying incoherence in speech: an automated methodology and novel application to schizophrenia. *Schizophr. Res.* **93**, 304–316 (2007). 17433866 10.1016/j.schres.2007.03.001 PMC1995127

[62] Boyd, R. L., Blackburn, K. G. & Pennebaker, J. W. The narrative arc: revealing core narrative structures through text analysis. *Sci. Adv.* **6**, eaba2196 (2020). 32821822 10.1126/sciadv.aba2196 PMC7413736

[63] Boyd, R. L. & Schwartz, H. A. Natural language analysis and the psychology of verbal behavior: the past, present, and future states of the field. *J. Lang. Soc. Psychol.* **40**, 21–41 (2021). 34413563 10.1177/0261927x20967028 PMC8373026

[64] Brooks, M. E. et al. glmmTMB balances speed and flexibility among packages for zero-inflated generalized linear mixed modeling. *The R Journal* **9**, 378–400 (2017).

[65] Bendetowicz, D., Urbanski, M., Aichelburg, C., Levy, R. & Volle, E. Brain morphometry predicts individual creative potential and the ability to combine remote ideas. *Cortex* **86**, 216–229 (2017). 27919546 10.1016/j.cortex.2016.10.021

[66] Teige, C. et al. Dynamic semantic cognition: characterising coherent and controlled conceptual retrieval through time using magnetoencephalography and chronometric transcranial magnetic stimulation. *Cortex* **103**, 329–349 (2018). 29684752 10.1016/j.cortex.2018.03.024 PMC6002612

[67] Ratcliff, R. Modeling response signal and response time data. *Cogn. Psychol.* **53**, 195–237 (2006). 16890214 10.1016/j.cogpsych.2005.10.002 PMC2397556

[68] Warr, R. L. & Collins, D. H. A comprehensive method for solving finite-state semi-Markov processes. *Int. J. Simul. Process Model.* **10**, 89 (2015).

[69] Eldar, E., Bae, G. J., Kurth-Nelson, Z., Dayan, P. & Dolan, R. J. Magnetoencephalography decoding reveals structural differences within integrative decision processes. *Nat. Hum. Behav.* **2**, 670–681 (2018). 31346283 10.1038/s41562-018-0423-3

[70] Coulthard, M. *An Introduction to Discourse Analysis* (Routledge, 2014); 10.4324/9781315835884

