# Supplementary material for: Latent mechanisms of language disorganization relate to specific dimensions of psychopathology
Source: Nat Ment Health. 2024 Nov 25;2(12):1486–97. doi: 10.1038/s44220-024-00351-w (PMC11621019; doi:10.1038/s44220-024-00351-w)
Supplement: Supplementary file 1 — Supplementary Figs. 1–9, Tables 1–8, Results 1–10 and Methods 1–3. [file 44220_2024_351_MOESM1_ESM.pdf]

nature mental health

Supplementary information https://doi.org/10.1038/s44220-024-00351-w

# Latent mechanisms of language disorganization relate to specific dimensions of psychopathology

In the format provided by the authors and unedited

**Supplementary Material**

**Supplementary Results 1.**

As noted in the main text, and demonstrated in Table S1, the relationship between self-reported Eccentricity and different measures of alterations in language and thought remained significant when controlling for key demographic and cognitive variables.

**Table S1 - Partial correlations of key variables with Eccentricity (& confidence intervals) after controlling for demographic variables, and verbal working memory**

| **Variable** | Age | Education | Only UK-based education | Only man | Only woman | Digit span (forward) | Digit span (backward) | All variables (linear regression) |
|---|---|---|---|---|---|---|---|---|
| Narrative Typicality | -.18 [-.25, -.13] | -.16 [-.22, -.10] | -.16 [-.22, -.10] | -.16 [-.26,. -06] | -.12 [-.20,. -04] | -.17 [-.23, -.11] | -.17 [-.23, -.11] | -.15 [-.21, -.10] |
| Narrative Coherence (5 words) | -.18 [-.24, -.12] | -.17 [-.24, -.12] | -.18 [-.24, -.12] | -.21 [-.31, -.11] | -.15 [-.23, -.07] | -.18 [-.24, -.12] | -.18 [-.24, -.12] | -.18 [-.23, -.11] |
| Mean Association Typicality | -.12 [-.18, -.05] | -.10 [-.17, -.04] | -.11 [-.17, -.05] | -.11 [-.21, -.003] | -.10 [-.18, -.02] | -.11 [-.17, -.05] | -.11 [-.17, -.05] | -.11 [-.17, -.04] |
| Replication - Association Typicality – no RS | -.20 [-.29, -.10] | -.18 [-.27, -.08] | -.23 [-.32, -.12] | -.20 [-.34, -.04] | -.18 [-.30, -.05] | -.18 [-.28, -.09] | -.18 [-.28, -.09] | -.18 [-.28, -.09] |
| Replication - Association Typicality – RS | -.19 [-.28, -.09] | -.17 [-.27, -.08] | -.23 [-.32, -.13] | -.19 [-.34, -.04] | -.18 [-.30, -.05] | -.18 [-.27, -.08] | -.18 [-.27, -.08] | -.19 [-.29, -.09] |
| Best-fitted β value | -.13 [-.19, -.07] | -.12 [-.18, -.06] | -.13 [-.20, -.07] | -.15 [-.25, -.03] | -.08 [-.15, .007] | -.12 [-.18, -.06] | -.12 [-.18, -.06] | -.11 [-.17, -.05] |
| Best-fitted $\alpha_l$ value | -.08 [-.14, -.02] | -.07 [-.13, -.01] | -.08 [-.14, -.01] | -.08 [-.18, .02] | -.04 [-.12, .03] | -.07 [-.14, -.01] | -.07 [-.14, -.01] | -.06 [-.12, .0008] |

Note. RS = response signal.

**Supplementary Results 2. Tangentiality, narrative length, and frequency in language**

As reported in the main paper, we found that people with high Eccentricity exhibit greater atypicality in the second ($r(998) = -.16$, $p < .001$), compared to the first half of their narratives ($r(998) = -.11$, $p < .001$), with a significant interaction ($\beta = 0.08$, $SE = 0.04$, $Z = 2.03$, $p = .042$). Notably, people with high self-reported Eccentricity also produced longer narratives ($r$ (998) = -.07, $p = .03$), suggesting that the increase in atypicality over time might be a by-product of greater elaboration. However, this was not supported by the data, as Eccentricity predicted greater atypicality in the second half of the narratives, even when controlling for narrative length ($\beta = 0.08$, $SE = 0.04$, $Z = 2.03$, $p = .042$). Furthermore, although people high in the Disorganized Thought dimension also produced longer narratives ($r$ (998) = -.08, $p = .01$), their narratives were not atypical (see Figure 3 in the main paper).

We also tested whether narrative atypicality in Eccentricity reflects the use of words that are less constrained (i.e., less common) by their specific context, rather than words that are simply infrequent in language (log-transformed frequency derived from the SUBTLEX-UK corpus). For instance, the words ‘Country’ and ‘Anthropomorphize’ are both very atypical in the context of the narratives participants were asked to tell (such that using them reflects a difficulty focusing on the topic in hand), yet differ in their frequency in language. Conversely, a word like ‘Conundrum’ or ‘Nefarious’ are relatively infrequent in English yet can be directly connected to the story of Cinderella.

This analysis showed that although Eccentricity predicted the use of less frequent words ($r$ (998) = -.14, $p < .001$) the association between Eccentricity and narrative atypicality remained significant, albeit weakened, even after controlling for general frequency ($r$ (998) = -.13, $p < .001$), or removing infrequent words (i.e., bottom 15%) from the narratives ($r$ (998) = -.14, $p < .001$). Further support for the idea that Eccentricity predicts the use of words that are less frequent specifically in the context in which they were produced is evident in the fact that when typicality was measured across probes (i.e., how similar is a narrative produced in response to one probe to the narratives all other participants produced in response to the other probe), it no longer correlated with Eccentricity ($r$ (998) = -.01, $p = .63$).

**Supplementary Results 3. Global and local coherence.**

As reported in the main paper, higher Eccentricity was associated with less coherent narratives, as measured by the cosine similarity between adjacent semantic expressions. Importantly, cosine similarity between adjacent semantic expressions reflects both the degree to which all different semantic expressions in the narrative reflect a single, coherent topic (global dispersion), and the degree to which the semantic expressions are organized in a coherent sequential order (local disorganization). To dissociate between local and global incoherence we examined the sensitivity of our results to shuffling the order of the semantic expressions (violin plots). To the extent that reduced cosine similarity specifically reflects local disorganization, we would expect shuffling to destroy the correlation between Eccentricity and cosine similarity. Our findings (Figure S1) indicate that although Eccentricity was mainly related to global incoherence, this did not fully explain the effects of local incoherence, in that the correlations between Eccentricity and cosine similarity were stronger without shuffling, for medium-sized semantic expressions.

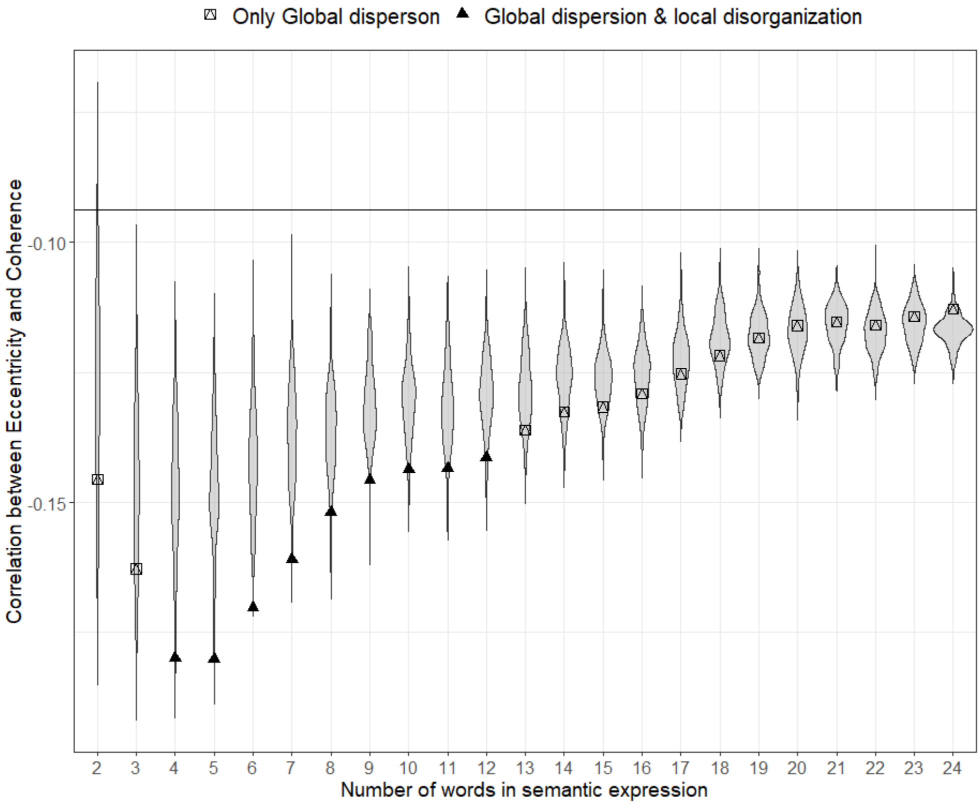


**Figure S1.** Local (filled triangles) and Global (see violin plots) semantic coherence. Here, filled triangles correspond with cases in which the correlation between Eccentricity and coherence is stronger than what might be expected in the case of global dispersion alone (the latter measured by shuffling the order of semantic expressions)

**Supplementary Results 4. Correlations among different measures of language and thought.**
As demonstrated in Table S2, the two main tasks used in the study (Free narrative writing and free association) were correlated, thus providing support for convergent validity.

**Table S2 - Correlations among different measures of language and thought**

| Variable | Narrative Typicality | Narrative Coherence (5 words) |
|---|---|---|
| Narrative Typicality | - | - |
| Narrative Coherence (5 words) | .37 [.32, .43] | - |
| Mean Association Typicality | .18 [.12, .24] | .06 [-.01, .12] |
| Best-fitted β value | .24 [.18, .30] | .06 [-.003, .12] |
| Best-fitted $\alpha_l$ value | .21 [.14, .26] | .10 [.04, .16] |
| Best-fitted $\alpha_{sign}$ value | .20 [.14, .26] | .08 [.01, .14] |

**Supplementary Results 5. Model fit, model comparison and parameter recovery.**
Prior to examining the correlations of model parameters with dimensions of psychopathology, we verified that the semi-Markov process (SMP) model can explain the data, fits better than alternative models, and produces recoverable parameters.

The results of this analysis suggested that the SMP could explain the distributions of speed and typicality of the reported associations at a group level and individual differences in average speed and typicality (Extended Data Figure 2). Furthermore, formal model comparison (Table S3) supported the assumption that participants do not always report the very first association that comes to mind, evident in the fact that models where people never reject associations perform worse in explaining the data. Crucially, examining model recovery has shown that the fitting procedure can reliably distinguish a model with no rejections (Model 6) from a model where α was free to vary (Model 2) with 100% accuracy, supporting the validity of the conclusion that participants used executive control to reject unsuitable (i.e., atypical) associations.

Conversely, the model comparison results pertaining to the auxiliary parameters scaling the RT distributions (namely λ and Eμ) showed a bias towards parsimony. Thus, a model with fixed λ did not only better explain data simulated from itself (85.71%), but also data simulated from a model with free λ (76.92%). Similarly, a model with fixed Eμ was better at explaining the data (100%) regardless of whether it was simulated from a model with free or fixed Eμ. Crucially, these model recoverability issues do not affect the study's conclusions, as demonstrated by the fact that the correlations

between key parameters ($\beta$ and $\alpha_l$) and psychopathological dimensions were equivalent regardless of the status of the auxiliary parameters (Table S3, Rows 1-4).

In addition, as shown in Table S4, satisfactory recoverability was found for $\beta$ and $\alpha_l$, with considerably lower recoverability for $\alpha_{sign}$. The latter results from the fact that deliberate creativity was almost non-existent in our sample (i.e., the probability that a participant mostly rejects weak associations was above 50% in 961/964 participants, and recovered parameters were based on fitted hyper-priors). Critically, the results for the other two parameters were similar when fixing $\alpha_{sign} = 1$ (Supplementary Results 1.6). Furthermore, the dominance of the correlation between Eccentricity and $\beta$ reported in the main text remained even when controlling (using the Spearman correction for attenuation) for differences in recoverability ($r_{(\beta,eccentric).\alpha l}(962) = -0.11$; $r_{(\alpha l,eccentric).\beta}(962) = -0.004$).

Finally, in addition to examining the recoverability of the fitted parameters, we examined whether the model recovers the distributions of associations across the entire sample, on which the model was based. This was examined by generating ‘simulated norms’ by aggregating the responses simulated based on best-fitted parameter values. This analysis suggested a significant difference between the empirical and simulated pTP distribution in only 6.6% of the simulations, just slightly above the significance level of 5%, thus confirming the ability of the model to account for the empirical norms across participants.

**Table S3 – Model comparison**

| **Model** | | | | | **Correlations with psychopathology** | | | |
|---|---|---|---|---|---|---|---|---|
| $\alpha_l$ | Eµ | λ | iBIC | Iter. | $r_{(\beta,eccentric).\alpha l}$ | $r_{(\alpha l,eccentric).\beta}$ | $r_{(\beta,suspicious).\alpha l}$ | $r_{(\alpha l,eccentric).\beta}$ |
| Free | Fixed to 0 | Fixed to 1 | 278979.7 | 9 | -.09** | -.05 | -.08* | .10** |
| Free | **Fixed to 0** | **Free** | **278929.6** | **12** | **-10**** | **-.03** | **-.09**** | **.12**** |
| Free | Free | Fixed to 1 | 279108.4 | 13 | -.09** | -.05 | -.09** | .09** |
| Free | Free | Free | 279012.8 | 15 | -.08** | -.04 | -.08* | .10** |
| No reject | Fixed to 0 | Fixed to 1 | 279285.2 | 10 | N/A | N/A | N/A | N/A |
| No reject | Fixed to 0 | Free | 279234.0 | 8 | N/A | N/A | N/A | N/A |
| No reject | Free | Fixed to 1 | 279374.2 | 8 | N/A | N/A | N/A | N/A |
| No reject | Free | Free | 279135.5 | 13 | N/A | N/A | N/A | N/A |

**Note.** A model with fixed $\alpha_i$ (title ‘No reject’) entails a model where no associations are rejected (i.e., a simple Gamma distribution, with a mean being a function of typicality). A model with $\lambda$ that is fixed to 1 entails an Exponential thinking-time distribution (instead of a Gamma distribution). The $\mu E$ parameter allows specifying a non-linear (i.e., power-law) function linking -log(p[Ai]) to the mean generation time. Iter – number of iterations until convergence of group-level priors. The highlighted model had the best fit and was used to obtain parameter estimates in the paper. Correlations with psychopathology correspond with partial Pearson correlations, and their two-sided (non-corrected) significance (* p < .05; ** p < .01)

| | **Table S4 - Parameter Recovery** | | | | | | |
|---|---|---|---|---|---|---|---|
| | β | Sμ | λ | $\alpha_l$ | $\alpha_{sign}$ | $\tau_0$ | $\tau_r$ |
| B | **0.69** | -0.12 | -0.10 | 0.23 | 0.35 | -0.01 | -0.07 |
| Sμ | -0.09 | **0.55** | 0.24 | 0.33 | 0.23 | 0.04 | 0.15 |
| λ | -0.03 | 0.10 | **0.22** | 0.08 | 0.07 | -0.11 | -0.04 |
| $\alpha_l$ | 0.15 | 0.29 | 0.16 | **0.50** | 0.32 | -0.02 | 0.08 |
| $\alpha_{sign}$ | 0.18 | 0.15 | 0.08 | 0.23 | **0.32** | 0.00 | 0.05 |
| $\tau_0$ | -0.02 | 0.05 | -0.33 | -0.01 | -0.01 | **0.79** | 0.18 |
| $\tau_r$ | -0.08 | 0.25 | -0.17 | 0.14 | 0.09 | 0.19 | **0.81** |

**Supplementary Results 6. Modeling results assuming that only atypical associations can be rejected.**

In the main paper, correlations with $\alpha_l$ (e.g., with Eccentricity) were examined regardless of whether a participant was more likely to reject typical or atypical association. This can be justified by the fact that the best-fitted values of $\alpha_{sign}$ indicating deliberate rejection of strong associations was very rare (the probability that a participant mostly rejects weak associations was above 50% in 961/964 participants). Yet, to ensure that the executive dysregulation in Eccentricity and executive over-regulation in Suspiciousness indeed reflect abnormal regulation of *atypical* associations, we repeated the analyses when assuming that only weak associations are rejected. In particular, since best-fitted parameters were estimated by averaging posterior samples, this could be achieved by only sampling posterior samples wherein $\alpha_{sign}$ =1 (86.92% of samples on average across participants; IQR = [80.54%, 95.81%]). As expected, this analysis has replicated the correlations with Eccentricity ($r_{(\beta,eccentric).\alpha l}$ (962) = -0.10, $p$ = .002; $r_{(\alpha l,eccentric).\beta}$ (962) = -0.01, $p$ = .60), and Suspiciousness ($r_{(\beta,suspiciousness).\alpha l}$ (962) = -0.10, $p$ = .002; $r_{(\alpha l, suspiciousness).\beta}$ (962) = 0.12, $p$ < .001).

**Supplementary Results 7. An experimental manipulation reducing executive regulation.**

To further establish under-constrained associative maps as a key mechanism for atypical language in self-reported Eccentricity, we investigated association atypicality after experimentally minimizing executive regulation of associations. Thus, in a pre-registered follow-up study (https://aspredicted.org/tmzc-xyft.pdf), 401 of our previous participants (in the condition they did not fail any attention check in the main study) were asked to repeat the Color Association Task with an additional condition discouraging the rejection of associations.

In this task version, participants were generally required to press the space bar once they had thought of an association, a method previously shown to reduce artefacts resulting from typing speed variability[1,2]. More importantly, in approximately half of trials, a response signal was presented urging

participants to start writing an association as quickly as possible (Participants had 3000ms to start typing a response after a response signal was presented). The latency of the response signal on each trial was determined by taking a random fraction of a randomly sampled RT from the participant's history, but it was never less than 300ms. This procedure ensures a broad yet personalized range of latencies. In addition, an adaptive procedure was used to verify that the proportion of trials with no response signal approaches 50%. Note also that, to screen for objective (rather than only self-reported) color blindness, the follow-up study started with fifteen plates from the Ishihara color blindness test[3], and only participants scoring above 90% were allowed to participate.

The key principles behind this manipulation are demonstrated in Extended Data Figure 3. If participants with high self-reported Eccentricity exhibit deliberate creativity by endeavouring to reject typical associations, this condition should act to constrain this tendency. Relatedly, even under an executive dysregulation account (i.e., people with high Eccentricity are equally likely to come up with a weak association, but are less likely to reject it), response signal trials are expected to reduce regulation across all participants, and thus eliminate individual differences in typicality. Conversely, if associative thinking is naturally more atypical and under-constrained, introducing a response signal should not eliminate the effect.

To generate formal predictions for each hypothesis for the follow-up study, we first estimated a new parameter value (one parameter per hypothesis) for each participant, based on its ability to predict the empirical average typicality. Thus, to simulate isolated predictions for the less-constrained associative maps hypothesis we estimated the $\beta$ parameter, while fixing $\alpha_l$ to the sample mean, and $\alpha_{sign}$ to 1. Similarly, for the executive dysfunction hypothesis, we estimated the $\alpha_l$ parameter while fixing β to the sample mean, and $\alpha_{sign}$ to 1. Finally, for the deliberate creativity hypothesis, we fixed $\alpha_{sign}$ to -1, and estimated the $\alpha_l$ parameter whereas $\beta$ was fixed to the sample average. Next, data for standard and response signal trials was simulated (200 simulations per participant) based on these estimated parameter values. The formal predictions presented in Extended Data Figure 3 are based on these simulations, and show that the `less-constrained associative maps` hypothesis alone would decrease typicality in response signal trials.

Finally, power analysis for each hypothesis was conducted by generating a simulated `Eccentricity` variable, with a correlation of -0.14 with the respective parameter (separately for each simulation). Then, either 100, 200, 300 and 400 participants were sampled from this simulated population, while oversampling the lower and upper end of this simulated Eccentricity variable (similar to how sampling in the actual replication study was conducted). For each simulated data set we calculated the power for an interaction effect (response signal X simulated Eccentricity), as well as the power for the respective simple effects (expecting an interaction under executive dysfunction and deliberate creativity, and two simple effects without an interaction under less-constrained associative maps).

The results of this analysis (Table S5) show that at least 400 participants are needed to attain sufficient power to detect the effect of less constrained associative maps, as well as the effect of an executive dysfunction. Conversely, note that the deliberative creativity hypothesis did not create a strong enough effect in either condition. The reason for this is that given that the sample mean value

of β was negative (i.e., ~ -0.2), atypical associations are prevalent even without high levels of deliberate creativity.

| | Table S5 – Response Signal Color Association Task power analysis results | | | | |
|---|---|---|---|---|---|
| **N** | | No response signal – negative effect | Response signal reached – negative effect | Response signal reached – positive effect | Interaction |
| 400 | Less-constrained associative maps | **93.0%** | **71.0%** | 0% | 15.5% |
| | Executive dysfunction | **91.0%** | **0%** | 0.1% | **80.0%** |
| | Deliberate creativity | 18.0% | **0%** | 0.2% | **10.0%** |
| 300 | Less-constrained associative maps | 65.0% | 33.0% | 0% | 9.5% |
| | Executive dysfunction | 56.0% | 0% | 0% | 50.0% |
| | Deliberate creativity | 15.0% | 0% | 0% | 10.5% |
| 200 | Less-constrained associative maps | 50.0% | 30.0% | 0% | 8.0% |
| | Executive dysfunction | 50.0% | 0% | 0% | 30.0% |
| | Deliberate creativity | 10.0% | 0% | 0% | 8.0% |
| 100 | Less-constrained associative maps | 32.0% | 12.0% | 0% | 8.0% |
| | Executive dysfunction | 22.0% | 0% | 0% | 16.0% |
| | Deliberate creativity | 10.0% | 0% | 0% | 6.0% |

The assumption that introducing a response signal reduces regulation of thought expression was supported by a finding that associations in such trials were less typical (β = -0.03, SE = 0.01, Z = -4.45, $p < .001$). More importantly, people with high Eccentricity produced more atypical associations (β = -0.04, SE = 0.01, Z = -4.80, $p < .001$) irrespective of whether a response signal had been presented or not (Extended Data Figure 3; both simple effects are significant; *p*'s < .001), with no significant interaction (β = 0.001, SE = 0.006, Z = 0.24, $p = .809$).

**Supplementary Results 8. Coherence in all psychiatric dimensions.**

As noted in the main text, only Eccentricity predicted lower coherence between adjacent semantic expressions (Figure S2)

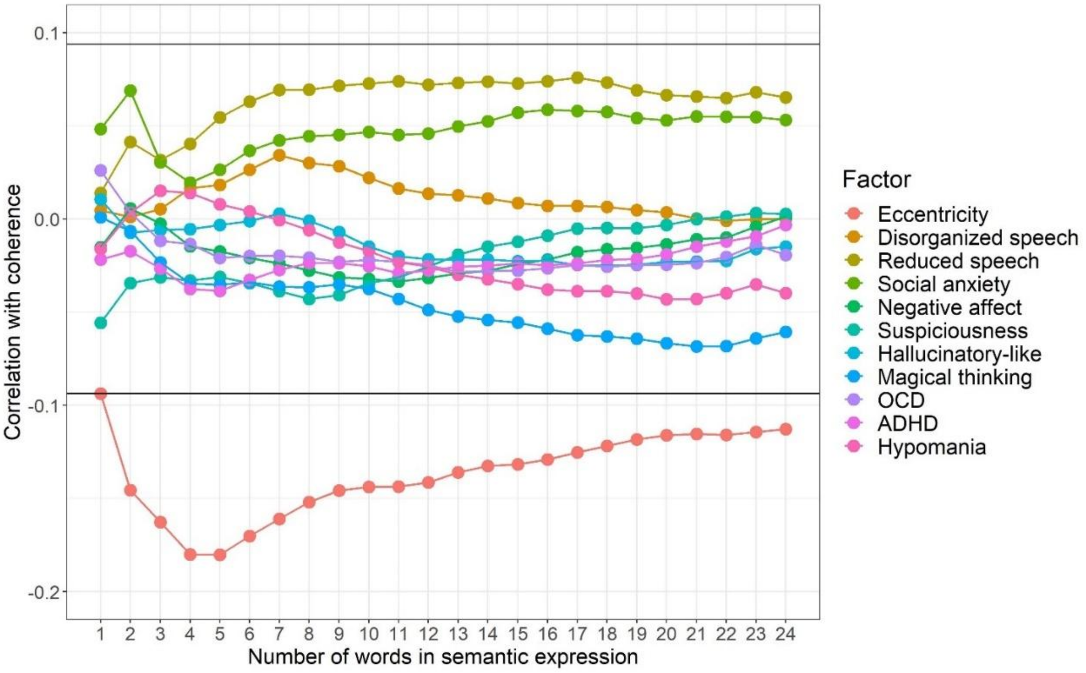


**Figure S2**. This figure complements Figure 5A in the main paper, in showing that only Eccentricity predicted lower narrative coherence. The horizontal line corresponds with the FDR-corrected significance criterion.

**Supplementary Results 9. Canonical correlation analysis.**

One way to determine the profile of associative thinking alterations in psychopathology involves defining latent dimensions of psychopathology based on their purported underlying computational mechanisms. For this purpose, we employed sparse canonical correlation analysis, which concurrently extracts latent dimensions of psychopathology (based on questionnaire items) and mechanisms (based on the two key computational parameters) with the goal (objective function) of maximizing their correlation[4–7]. We used an established nested cross-validation procedure to avoid over-fitting[8]. Specifically, we used 5-fold cross validation which extracts five independent test sets used for calculating the canonical correlations after training the model on the complementary training sets. The L1-regularization hyperparameter was tuned using a separate 3-fold cross validation only within the training sets (averaging across 10 random repetitions of the 3-fold cross validation to reduce variance). Note that regularization was used only for the questionnaire items but not for the computational parameters (also note that an additional, maximal L2 regularization is used in this algorithm[5]). This procedure was applied for finding the first latent (canonical) variables. The significance of the correlation between these variables was tested using a permutation test, such that, each time (5000 permutations overall) the sCCA algorithm is trained using a permuted training set, with the optimal regularization parameter found in the original training set (see above), and then applied to the (non-permuted) test set[8]. Since the first canonical correlation was significant, we continued to examine the second set of latent (canonical) variables and their correlation after

‘projection deflation’[8], which is a method that removes the first set of latent variables from the questionnaire scores and computational parameters.

The results of this analysis provided further support for our finding of involvement of overt and covert thought disorganization in distinct dimensions of psychopathology (Extended Data Figure 4). Thus, whereas increased self-reported Eccentricity in speech and behavior (but also, interestingly, some hypomanic symptoms) and reduced social anxiety predicted less-constrained associative maps and executive dysregulation, difficulties trusting and confiding in other people predicted less-constrained associative maps with increased regulation of speech.

**Supplementary Results 10. Psychiatric dimensions and social functioning**

Many psychiatric dimensions correlated with loneliness (as inversely measured by the Friendship Scale[9]), and social anhedonia (inversely measured by the Interpersonal Pleasure Scale[10]; see Table S6). Critically, however, relative importance analysis based on a multiple linear regression has shown that Eccentricity and Suspiciousness are the most important variables explaining social anhedonia, and among the three most important variables explaining loneliness (the third being Negative Affect; Figure S3)

**Table S6 - Correlations between psychiatric dimensions and social functioning**

| | Friendship scale (FS) | Interpersonal pleasure scale |
|---|---|---|
| ACIPS | 46 [.38, .54] | |
| Negative Affect | -.61 [-.67, -.54] | -.28 [-.37, -.19] |
| OCD | -.42 [-.50, -.34] | -.18 [-.28, -.09] |
| **Reduced Speech** | **-.43 [-.51, -.35]** | **-.31 [-.39, -.21]** |
| ADHD | -.31 [-.40, -.22] | -.07 [-.17, .02] |
| Hallucinatory-like | -.41 [-.48, -.32] | -.05 [-.15, .04] |
| **Disorganized Speech** | **-.24 [-.33, -.15]** | **-.05 [-.15, .05]** |
| Hypomania | -.14 [-.23, -.04] | .15 [.05, .24] |
| Magical Thinking | -.26 [-.35, -.16] | .07 [-.03, .16] |
| **Social Anxiety** | **-.44 [-.51, -.35]** | **-.29 [-.37, -.19]** |
| **Eccentricity** | **-.43 [-.51, -.35]** | **-.33 [-.42, -.24]** |
| **Suspiciousness** | **-.50 [-.57, -.43]** | **-.47 [-.54, -.39]** |

**Table S7 - Correlations between psychiatric dimensions and social functioning after controlling for demographic variables**

| | Friendship scale (FS) | Interpersonal pleasure scale (ACIPS) |
|---|---|---|
| Negative Affect | -.59 [-.65, -.52] | -.30 [-.39, -.20] |
| OCD | -.40 [-.48, -.31] | -.19 [-.29, -.09] |
| **Reduced Speech** | **-.41 [-.49, -.33]** | **-.32 [-.40, -.22]** |
| ADHD | -.29 [-.38, -.20] | -.10 [-.19, -.00] |
| Hallucinatory-like | -.39 [-.47, -.30] | -.07 [-.16, .03] |
| **Disorganized Speech** | **-.24 [-.33, -.14]** | **-.07 [-.16, .03]** |
| Hypomania | -.11 [-.20, -.01] | .13 [.03, .23] |
| Magical Thinking | -.26 [-.35, -.16] | .07 [-.03, .16] |
| **Social Anxiety** | **-.41 [-.49, -.32]** | **-.31 [-.40, -.22]** |
| **Eccentricity** | **-.43 [-.51, -.35]** | **-.35 [-.43, -.26]** |
| **Suspiciousness** | **-.52 [-.59, -.44]** | **-.46 [-.54, -.38]** |

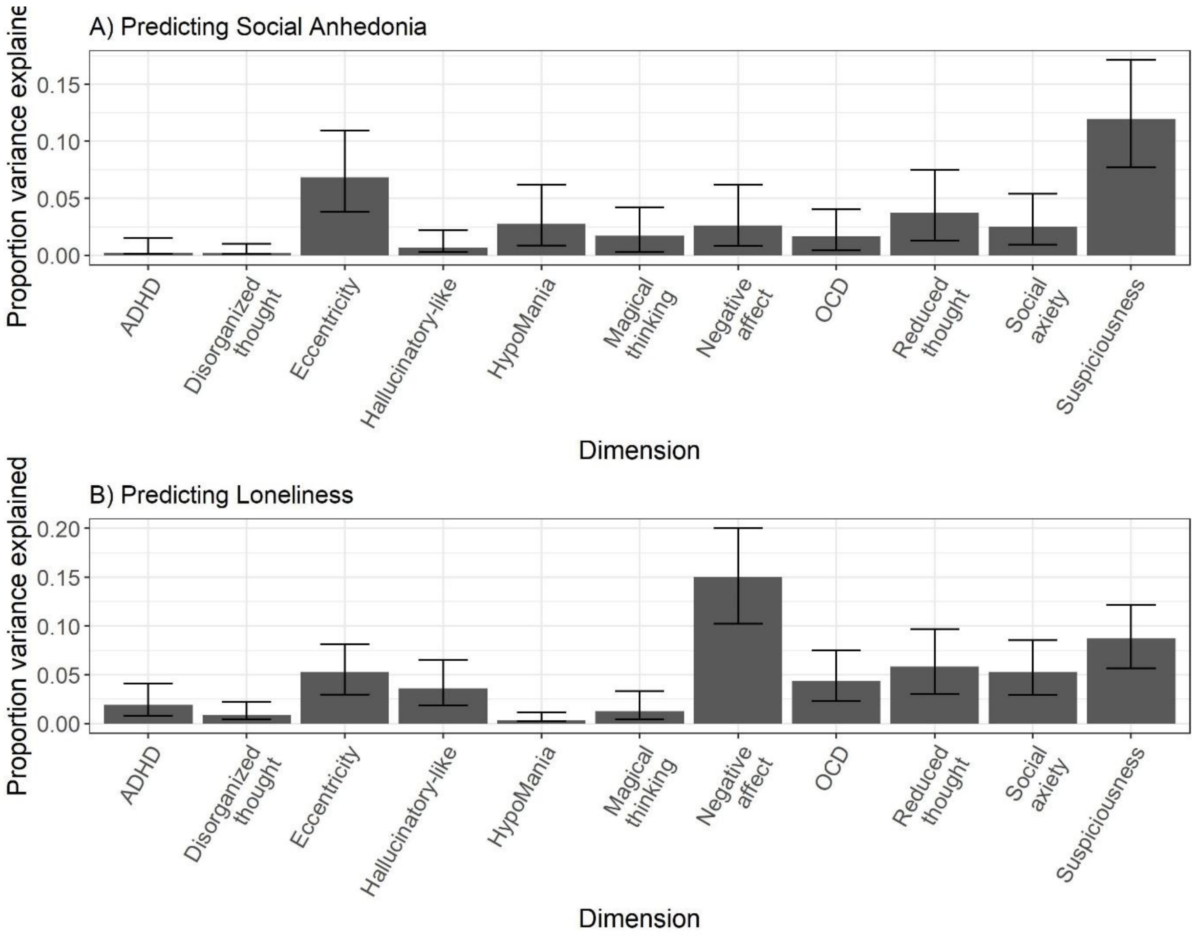


**Figure S3.** Relative importance (and bootstrapped 95% confidence intervals) of different psychopathological dimensions (N = 401) in predicting social anhedonia (A) and loneliness (B). Calculated using the R *relaimpo* package.

**Supplementary Methods 1. Participants, and inclusion and exclusion criteria.**

One thousand one hundred participants were recruited via the Prolific Academic internet platform, using the following inclusion criteria: Adults (from 18-70 years old), raised monolingual with English as a first language, currently residing in the United Kingdom, who did not report having color blindness, and had a minimum approval rate of 95% from at least 50 previous studies and at most 10,000 submissions. Participants failing one or more of five attention check questions distributed across the questionnaires (see Table S8) were excluded from further analysis. This led to the exclusion of one hundred participants (9.09%), in line with inattentive performance rates found in previous studies[11,12]. Additional three participants did not complete the Color association task, and additional two participants completing this task did not produce any appropriate responses (i.e., producing only misspelled words or single characters). Thus, analyses concerning individual differences in participants' performance in this task included 995 participants. An additional 31 participants had less than ten valid responses in this task and therefore were excluded from modeling analysis.

**Table S8 – Attention check questions, criteria, and excluded participants**

| Question | | N. Participants excluded |
|---|---|---|
| I enjoyed the music of Marlene Sandersfield | Moderately, A lot, Extremely | 16 |
| I like getting speeding tickets | Definitely agree | 2 |
| How often do you have difficulty remembering your name | Often, Very Often | 10 |
| I am thinking about the computer terms filtibly and proxypod | Sometimes, Quite often, Very often | 35 |
| I understand the meaning of the statements in this questionnaire | Almost never | 47 |
| Total (9 participants failed more than one attention check) | | 100 |

**Supplementary Methods 2. Free narrative analysis – additional details.**

As noted in the main text, the pre-processed narratives were then submitted to two types of natural language processing analyses. First, to measure narrative typicality, we calculated the similarity between each participant's narrative and the respective narratives of all other participants, under-weighting words repeating across all narratives (using term frequency-inverse document frequency weighting; tf-idf weighting; see Figure S4 below for an illustration).

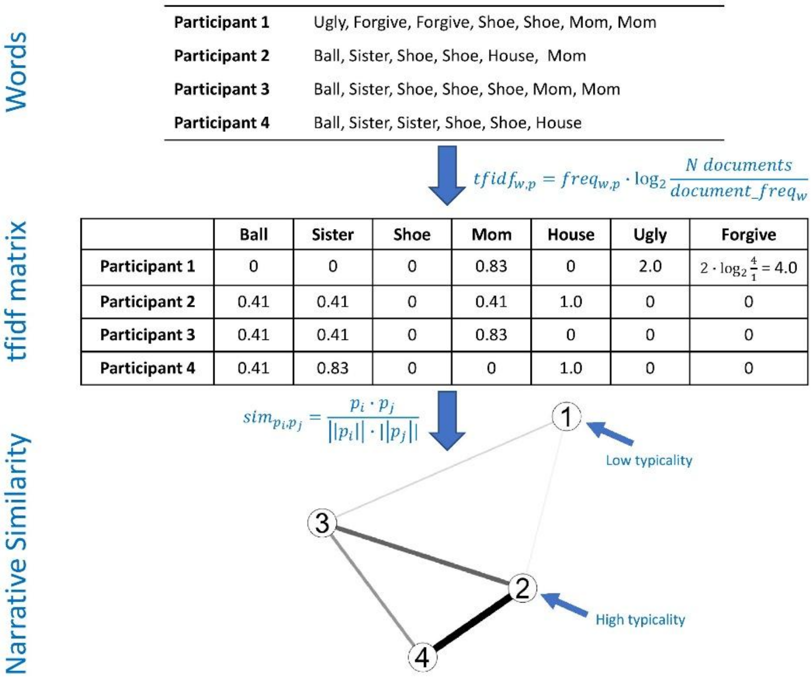


**Figure S4.** Illustration of the method used to calculate narrative typicality. Narratives are segmented into individual words (order indifferent). Then, each narrative is converted into a vector where each word is given a numeric value that is proportional to the frequency of each word in that narrative ($freq_{w,p}$), and inversely proportional to the percentage of narratives in which this word is included ($document\_freq_w$). Similarity between narratives is then computed as the cosine similarity (represented by both line thickness and line lengths in the bottom panel), whereas typicality is just the average similarity of a participant's narrative to the respective narratives of all other participants.

Second, to measure the internal coherence of the narratives, we examined the semantic similarity between consecutive words (or groups of words), using several popular embedding models. Figure 3B in the main text presents the coherence results using Word Mover's distance based on fastText embeddings, because this metric and this model produced an optimal combination of high shared variance between the two narratives, and relatively low correlation with the typicality measure specified above (Figure S5). Yet, as depicted in Figure S6, consistent results were found for other embedding models and metrics. In addition, Figure S7 depicts the correlations between the different embedding models and metrics.

Finally, we note that The first 234 participants were also asked to describe the classic 'Cookie theft' picture[13,14]. However, since the entire study (including questionnaires, three narratives and free association task) took more time than originally expected (i.e., more than an hour) we decided to remove this narrative from the experiment for all other participants. Our choice to remove this specific narrative was driven by the fact that it was less correlated (in terms of semantic coherence NLP metrics) with the other two narratives (Figure S8), thereby maximizing internal consistency[15]. Note, however, that the results for the first 234 participants, when including this narrative, were largely consistent with the results reported above (see Figure S9)

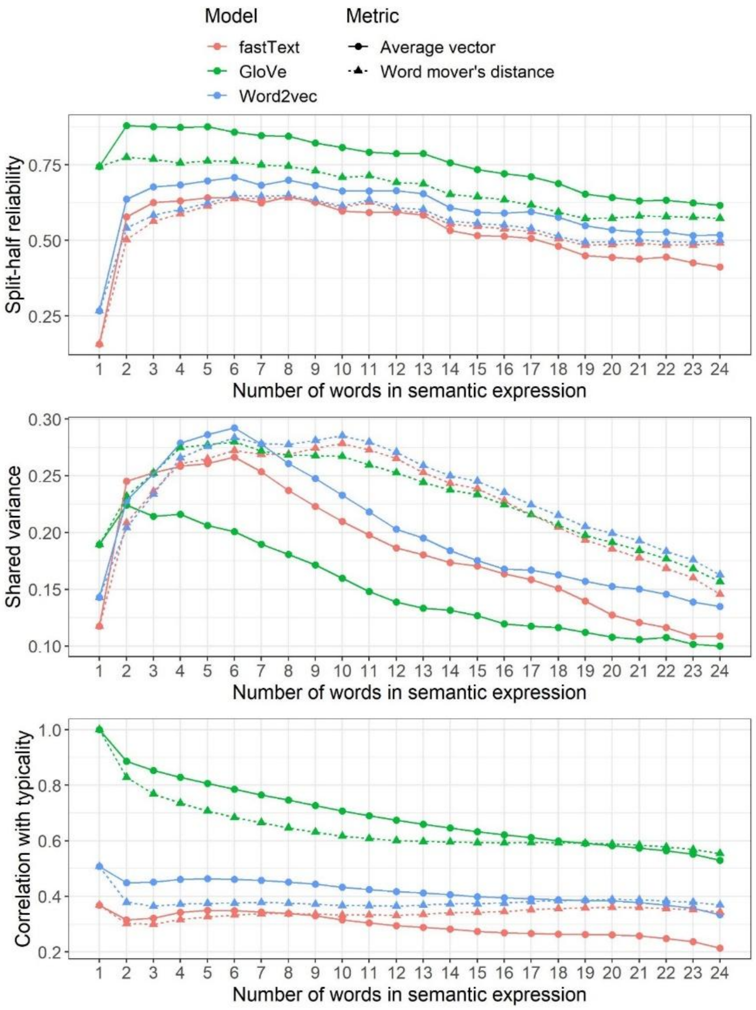


**Figure S5.** We compared three embedding models and two popular methods for extracting similarity between groups of words in terms of reliability, shared variance (between the two probes), and correlation with typicality. Word mover's distance based on fastText embeddings was used in the main analyses reported in the paper due to relatively high shared variance and low correlation with narrative typicality.

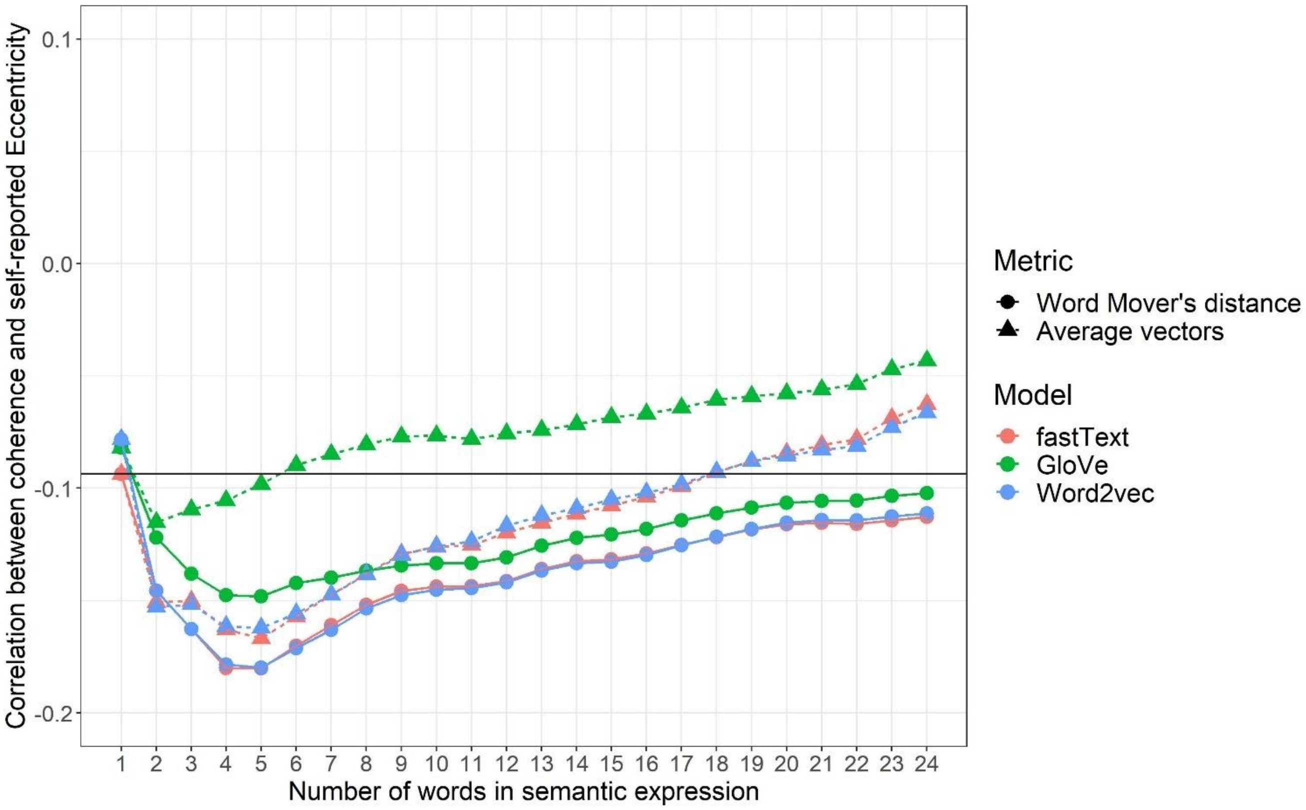


**Figure S6.** This figure complements Figure 3B in the main paper, in showing that Eccentricity predicted less coherent narratives even when using other embedding models and other metrics. The horizontal line corresponds with the FDR-corrected significance criterion.

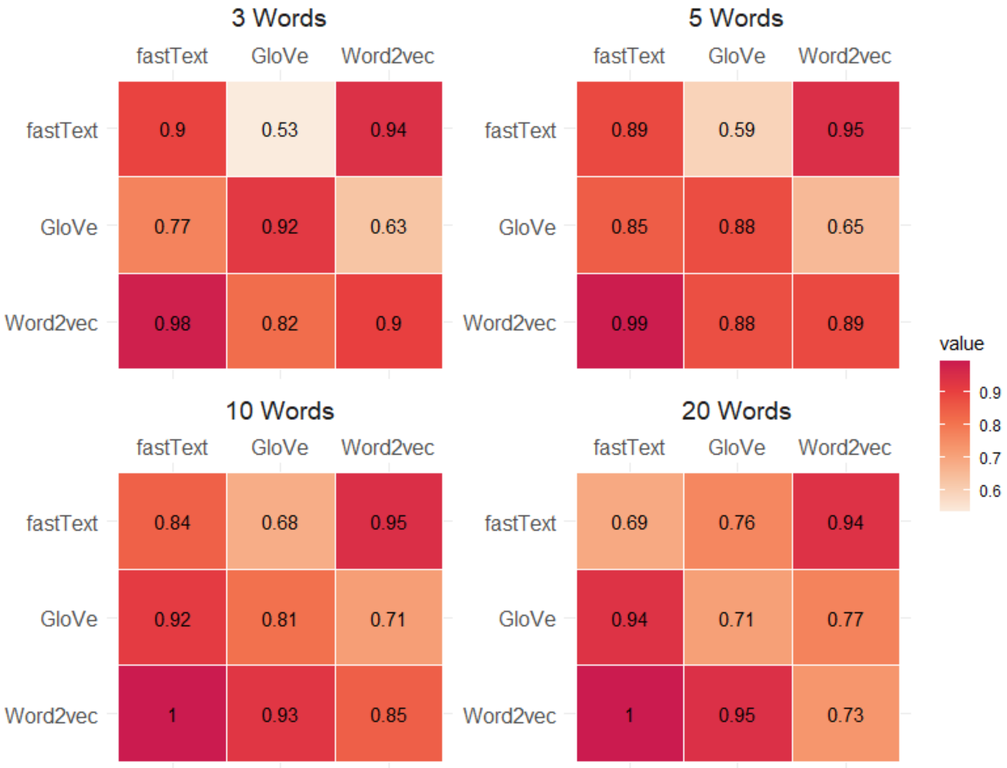


**Figure S7.** Correlations between different coherence metrics (word mover's distance and average vectors) calculated based on different embedding models. The diagonal represents the correlation between the two metric types (for a given model). The lower triangular corresponds with correlations between different embedding models representing groups of words by averaging the vectors of the individual words. The upper triangular corresponds with correlations between different embedding models wherein coherence between two groups of N words was calculated using the word mover's distance metric.

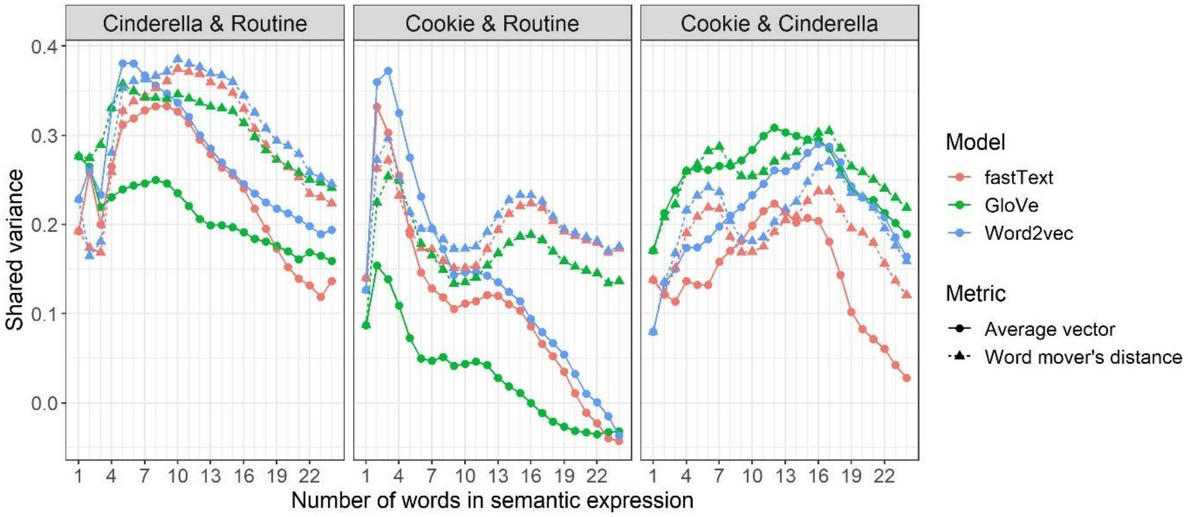


**Figure S8.** This figure justified our decision to exclude the additional narrative prompt asking participants to describe the 'cookie-theft' image, by the fact that its correlations with the other two narratives were relatively low. Note that these results are only based on the first 234 participants who were presented with this additional prompt (prior to its exclusion)

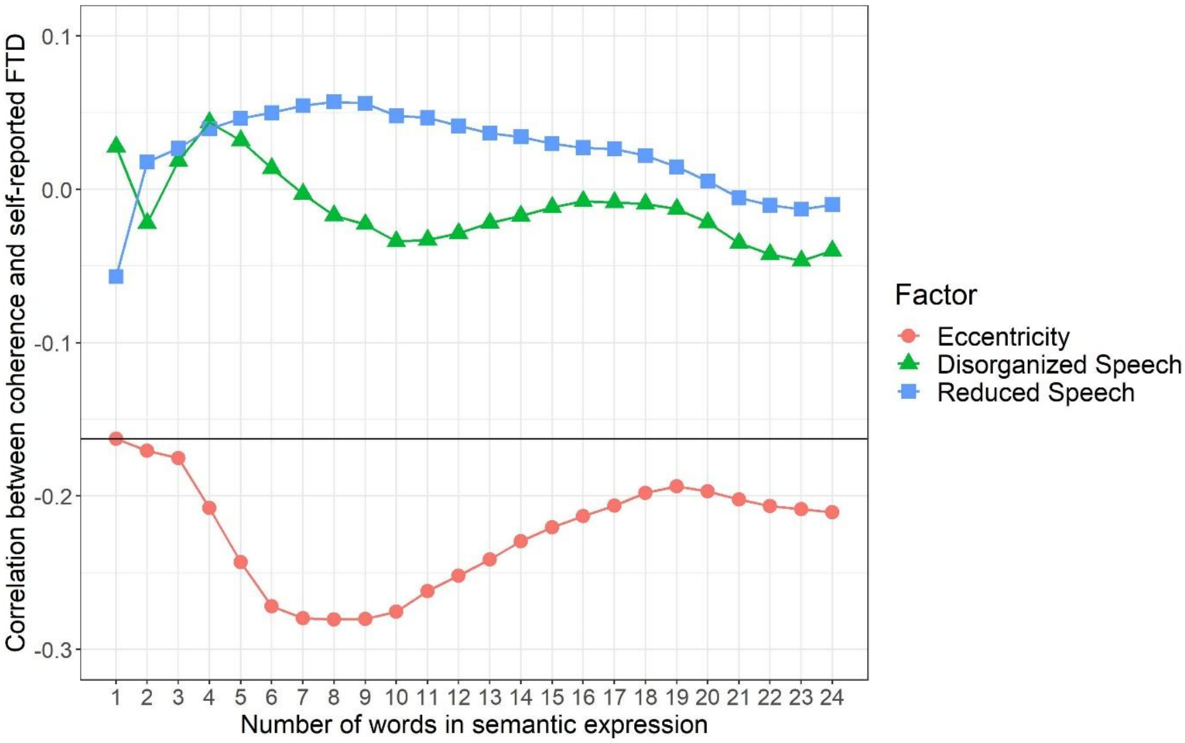


**Figure S9.** The figure shows that the main effect of Eccentricity was insensitive to the exclusion of the additional cookie-theft prompt, which was only presented to the first 234 participants, and then excluded due to relatively low correlations with the other two prompts (see Figure S8)

**Supplementary Methods 3. Semi-Markov Process Model – Hyperpriors for fitting.**

As noted in the main text, participant-level parameters were fitted using an iterative hierarchical expectation-maximization procedure, designed to compensate for the relatively small number of trials from each participant by using empirical group-level priors to constrain participant-level parameters. First, for each participant, we sampled 1000 different random settings of parameters from non-informative pre-defined group-level prior distributions:

$$\beta \sim Skewed\_Normal(0,2,0)$$
$$S\mu \sim Skewed_N ormal(0,2,0)$$
$$E\mu \sim Exponential(1)$$
$$\lambda \sim Exponential(90,81)$$
$$\alpha_I \sim Skewed\_Normal(0.5,0.4,0)$$
$$\alpha_{sign} \sim Bernoulli(0.5)$$
$$\tau_0 \sim Gamma(0.1,0.1)$$
$$\tau_r \sim Gamma(0.1,0.1)$$

A skewed-normal distribution was used for some parameters, to enable fitting of the empirical distributions of posterior parameters values (at each iteration) which were clearly negatively-skewed. For example, $\beta$ posteriors suggested that whereas most participants had a $\beta$ around 0, most of the variation was between participants with $\beta$ <0 values, with much less variation among the few participants with $\beta$ > 1 values. Note also that these group-level priors are non-informative within a reasonable scale of the respective parameter. For example, the prior for $\alpha_I$ ranges from -1 to 2, which mean, respectively, that either no associations are rejected, or that all associations are rejected.

## References


1. Fradkin, I. & Eldar, E. If you don't let it in, you don't have to get it out: Thought preemption as a method to control unwanted thoughts. *PLoS Comput. Biol.* **18**, e1010285 (2022).

2. Fradkin, I. & Eldar, E. Accumulating evidence for myriad alternatives: Modeling the generation of free association. *Psychol. Rev.* **130**, 1492–1520 (2023).

3. Ishihara, S. *Test for colour-blindness*. (dfisica.ubi.pt, 1987).

4. Xia, C. H. *et al.* Linked dimensions of psychopathology and connectivity in functional brain networks. *Nat. Commun.* **9**, 3003 (2018).

5. Witten, D. M., Tibshirani, R. & Hastie, T. A penalized matrix decomposition, with applications to sparse principal components and canonical correlation analysis. *Biostatistics* **10**, 515–534 (2009).

6. Haas, S. S. *et al.* Linking language features to clinical symptoms and multimodal imaging in individuals at clinical high risk for psychosis. *Eur. Psychiatry* **63**, e72 (2020).

7. Chapman, J. & Wang, H.-T. CCA-Zoo: A collection of Regularized, Deep Learning based, Kernel, and Probabilistic CCA methods in a scikit-learn style framework. *JOSS* **6**, 3823 (2021).

8. Monteiro, J. M., Rao, A., Shawe-Taylor, J., Mourão-Miranda, J. & Alzheimer's Disease Initiative. A multiple hold-out framework for Sparse Partial Least Squares. *J. Neurosci. Methods* **271**, 182–194 (2016).

9. Hawthorne, G. Measuring social isolation in older adults: development and initial validation of the friendship scale. *Soc. Indic. Res.* **77**, 521–548 (2006).

10. Gooding, D. C. & Pflum, M. J. The assessment of interpersonal pleasure: introduction of the Anticipatory and Consummatory Interpersonal Pleasure Scale (ACIPS) and preliminary findings. *Psychiatry Res.* **215**, 237–243 (2014).

11. Zorowitz, S., Niv, Y. & Bennett, D. Inattentive responding can induce spurious associations between task behavior and symptom measures. (2021) doi:10.31234/osf.io/rynhk.

12. DeSimone, J. A. & Harms, P. D. Dirty Data: The Effects of Screening Respondents Who Provide Low-Quality Data in Survey Research. *J. Bus. Psychol.* **33**, 559–577 (2018).

13. Gupta, T., Hespos, S. J., Horton, W. S. & Mittal, V. A. Automated analysis of written narratives reveals abnormalities in referential cohesion in youth at ultra high risk for psychosis. *Schizophr. Res.* **192**, 82–88 (2018).

14. Goodglass, H., Kaplan, E. & Weintraub, S. BDAE: The Boston Diagnostic Aphasia Examination. (2001).

15. Clark, L. A. & Watson, D. Constructing validity: Basic issues in objective scale development. Special Issue: Methodological issues in psychological assessment research. *Psychological Assessment* **7**, 309–319 (1995).
