# Supplementary material for: Latent mechanisms of language disorganization relate to specific dimensions of psychopathology
Source: Nat Ment Health. 2024 Nov 25;2(12):1486–97. doi: 10.1038/s44220-024-00351-w (PMC11621019; doi:10.1038/s44220-024-00351-w)
Supplement: Supplementary file 2 — Reporting Summary [file 44220_2024_351_MOESM2_ESM.pdf]

Corresponding author(s): Isaac Fradkin

Last updated by author(s): Oct 5, 2024

Reporting Summary

Nature Portfolio wishes to improve the reproducibility of the work that we publish. This form provides structure for consistency and transparency in reporting. For further information on Nature Portfolio policies, see our Editorial Policies and the Editorial Policy Checklist.

Statistics

For all statistical analyses, confirm that the following items are present in the figure legend, table legend, main text, or Methods section.

n/a	Confirmed	
☐	☒	The exact sample size (*n*) for each experimental group/condition, given as a discrete number and unit of measurement
☐	☒	A statement on whether measurements were taken from distinct samples or whether the same sample was measured repeatedly
☐	☒	The statistical test(s) used AND whether they are one- or two-sided<br>*Only common tests should be described solely by name; describe more complex techniques in the Methods section.*
☐	☒	A description of all covariates tested
☐	☒	A description of any assumptions or corrections, such as tests of normality and adjustment for multiple comparisons
☐	☒	A full description of the statistical parameters including central tendency (e.g. means) or other basic estimates (e.g. regression coefficient) AND variation (e.g. standard deviation) or associated estimates of uncertainty (e.g. confidence intervals)
☐	☒	For null hypothesis testing, the test statistic (e.g. *F*, *t*, *r*) with confidence intervals, effect sizes, degrees of freedom and *P* value noted<br>*Give P values as exact values whenever suitable.*
☐	☒	For Bayesian analysis, information on the choice of priors and Markov chain Monte Carlo settings
☐	☒	For hierarchical and complex designs, identification of the appropriate level for tests and full reporting of outcomes
☐	☒	Estimates of effect sizes (e.g. Cohen's *d*, Pearson's *r*), indicating how they were calculated

Our web collection on statistics for biologists contains articles on many of the points above.

Software and code

Policy information about availability of computer code

Data collection

Data was collected using using Gorilla Experiment Builder (Build-2021-32)

Data analysis

R version 4.1.3.
Python version 3.9.1.
Mplus v7

NLTK python library V3.8
Rcpp R package (version 0.8.3).
Hunspell R package V1.7.2
Textstem R package V0.0.1
GLMTMB R package V1.1.6

All code is available at:
https://osf.io/58k7y/

For manuscripts utilizing custom algorithms or software that are central to the research but not yet described in published literature, software must be made available to editors and reviewers. We strongly encourage code deposition in a community repository (e.g. GitHub). See the Nature Portfolio guidelines for submitting code & software for further information.

Data

Policy information about availability of data

All manuscripts must include a data availability statement. This statement should provide the following information, where applicable:

- Accession codes, unique identifiers, or web links for publicly available datasets
- A description of any restrictions on data availability
- For clinical datasets or third party data, please ensure that the statement adheres to our policy

Data used for analysis, including questionnaire scores, NLP measures derived from participants' narratives, and data from the color association task can be found in https://osf.io/58k7y/. Per study protocol and the consent form, participant's actual associations and narratives are immediately available upon request from the first author (Isaac Fradkin - itzik.fradkin@gmail.com).

Human research participants

Policy information about studies involving human research participants and Sex and Gender in Research.

Reporting on sex and gender	We collected only self-reported gender information. We did not collect sex information separately. Gender information is provided in the open data (see above), with participants` consent.
Population characteristics	See below
Recruitment	Participants were recruited via the Prolific Academic internet platform, using the following inclusion criteria: Adults (from 18-70 years old), raised monolingual with English as a first language, currently residing in the United Kingdom, who did not report having color blindness, and had a minimum approval rate of 95% from at least 50 previous studies and at most 10,000 submissions.<br><br>Key biases in internet studies such as ours concern the fact that all of our participants had minimal computer/internet skills to participate in online studies. We also deliberately recruited participants from the United Kingdom (based on the ethical approval) who stated that English is their first language - which might also limit the generalizability of our results
Ethics oversight	The study has been approved by the UCL research ethics committee (REC No 16639/001) and written informed consent was obtained from all participants

Note that full information on the approval of the study protocol must also be provided in the manuscript.

Field-specific reporting

Please select the one below that is the best fit for your research. If you are not sure, read the appropriate sections before making your selection.

☐ Life sciences ☒ Behavioural & social sciences ☐ Ecological, evolutionary & environmental sciences

For a reference copy of the document with all sections, see nature.com/documents/nr-reporting-summary-flat.pdf

Behavioural & social sciences study design

All studies must disclose on these points even when the disclosure is negative.

Study description	Mixed methods. The main study is quantitative cross-sectional, focusing on the correlations between self-reported, and behavioral data. The follow-up study is quantitative experimental, as it includes a specific manipulation designed to reduce executive regulation.
Research sample	A random sample of one thousand one hundred participants were recruited via the Prolific Academic internet platform, using the following inclusion criteria: Adults (from 18-70 years old), raised monolingual with English as a first language, currently residing in the United Kingdom, who did not report having color blindness, and had a minimum approval rate of 95% from at least 50 previous studies and at most 10,000 submissions. This number of participants was based on common guidelines concerning the required ratio of participants-to-questionnaire items for exploratory factor analysis, as well as previous transdiagnostic studies. 401 participants were recruited for an additional task based on a pre-registered power analysis.<br>We used this random convenience sampling technique to facilitate the recruitment of a large enough sample to capture transdiagnostic individual differences. We did not aim to recruit a representative sample, since our focus was on individual differences rather than accurately representing the general population.<br><br>Participants (in the final sample, see exclusions below) included 625 women (62.5%) and 360 men (36.0%) and were 38.22 (SD = 13.01) years old on average. The highest level of education acquired was high-school for 230 (23.0%) participants, a bachelor's degree for 381 participants (38.1%) and another higher-education diploma (i.e., level 4+ qualification) for 160 participants (16.0%); 211 participants had a master's degree or higher (21.1%; additional 16 participants did not finish upper secondary school and 2 did not disclose their level of education). Whereas participants were only allowed to participate if they reported English as their native

language, we also asked participants where they received their primary and secondary education to gain further insight into language proficiency. The results have shown that most participants received their primary and secondary education in the United Kingdom (95.1% and 96.1%, respectively) or another English-speaking country (2.8% and 1.9%, respectively).

Sampling strategy	We recruited a random convenience sample. The number of participants was based on common guidelines concerning the required ratio of participants-to-questionnaire items for exploratory factor analysis, as well as indications from previous trans-diagnostic studies (e.g., Wise, Robinson & Gillan, 2023). 401 participants were recruited for an additional task based on a pre-registered power analysis (https://aspredicted.org/tmzc-xyft.pdf), wherein effect sizes and power were calculated based on the first study results and model-based simulations.
Data collection	The task was administered over the internet using Gorilla Experiment Builder. Participants used their own computers (smartphones and tablets were not allowed). The researcher was blind to the within-subject study conditions, but not to the hypotheses. However, the latter does entail a risk of bias, since this completely online study included no direct contact between the researcher and the participants,
Timing	The basic sample was recruited from 16/11/2021-25/01/2022.<br>Then, 401 participants were invited to take part in a follow-up study including a novel task. This follow-up study (called Session 2 in the paper) was run from 11/03/2022 - 18/03/2022
Data exclusions	Participants failing one or more of five attention check questions distributed across the questionnaires were excluded from further analysis. This led to the exclusion of one hundred participants (9.09%), in line with inattentive performance rates found in previous studies. Additional three participants did not complete the Color association task, and additional two participants completing this task did not produce any appropriate responses (i.e., producing only misspelled words or single characters). Thus, analyses concerning individual differences in participants' performance in this task included 995 participants. An additional 31 participants had less than ten valid responses in this task and therefore were excluded from the modeling analysis.
Non-participation	This is difficult to estimate accurately in internet studies. 356 participants have returned the study, which means (in Prolific) that they intended to take part but either did not start or did not complete the study.
Randomization	Participants were not allocated to experimental groups. Dimensional measures of psychopathology were collected using self-reported questionnaires (and submitted to exploratory factor analysis, which inherently separates specific and general factors). Other relevant covariates were controlled across analyses (inc. age, gender, education, country of education, verbal working memory).

Reporting for specific materials, systems and methods

We require information from authors about some types of materials, experimental systems and methods used in many studies. Here, indicate whether each material, system or method listed is relevant to your study. If you are not sure if a list item applies to your research, read the appropriate section before selecting a response.

Materials & experimental systems

n/a	Involved in the study	
☒	☐	Antibodies
☒	☐	Eukaryotic cell lines
☒	☐	Palaeontology and archaeology
☒	☐	Animals and other organisms
☒	☐	Clinical data
☒	☐	Dual use research of concern

Methods

n/a	Involved in the study	
☒	☐	ChIP-seq
☒	☐	Flow cytometry
☒	☐	MRI-based neuroimaging
